# Updates to the Spectrum/AIM model for the UNAIDS 2020 HIV estimates

**DOI:** 10.1002/jia2.25778

**Published:** 2021-09-21

**Authors:** John Stover, Robert Glaubius, Reshma Kassanjee, Caitlin M. Dugdale

**Affiliations:** ^1^ Center for Modeling, Planning and Policy Analysis Avenir Health Glastonbury CT USA; ^2^ Centre for Infectious Disease Epidemiology and Research (CIDER) University of Cape Town Cape Town South Africa; ^3^ Division of Infectious Diseases Massachusetts General Hospital Boston MA USA

**Keywords:** CD4, COVID‐19, estimates, HIV, mortality, Spectrum

## Abstract

**Introduction:**

The Spectrum/AIM model is used by national HIV programs and UNAIDS to prepare annual estimates of key HIV indicators. This article describes key updates to paediatric and adult models for the 2021 round of HIV estimates.

**Methods:**

Potential updates to Spectrum arise due to newly available data, new analyses of existing data, and the need for new issues to be addressed. Updates are guided by experts through the UNAIDS Reference Group on Estimates, Modelling and Projections. Changes are tested and assessed for impact before being accepted into the final model.

**Results:**

Spectrum tracks children living with HIV by CD4% for ages 0–4 and CD4 count for ages 5–14. Data from IeDEA treatment sites have been used to map the transition from CD4% to CD4 count at age 5. Breastfeeding patterns in sub‐Saharan Africa have been updated with the latest survey data and estimates of continuation on antiretroviral therapy (ART) with breastfeeding have been revised based on recent studies. Model assumptions about the CD4 counts of people who drop out of ART have been revised to account for CD4 count increases while on treatment. If available, monthly data on numbers on ART can now be used to estimate the effects of COVID‐19‐related disruptions during 2020.

**Conclusions:**

These changes are intended to provide more accurate estimates of HIV burden. The effects of these changes on paediatric indicators are small except in countries with new surveys that might have updated patterns of breastfeeding. Changes to the adult model have little effect on total new infections. AIDS‐related deaths will be somewhat lower in countries that have data on ART drop out but might be increased by HIV care disruptions due to COVID‐19. The updated model uses newly available data to improve the estimation of paediatric and adult HIV indicators.

## 1 INTRODUCTION

**Figure 1:**
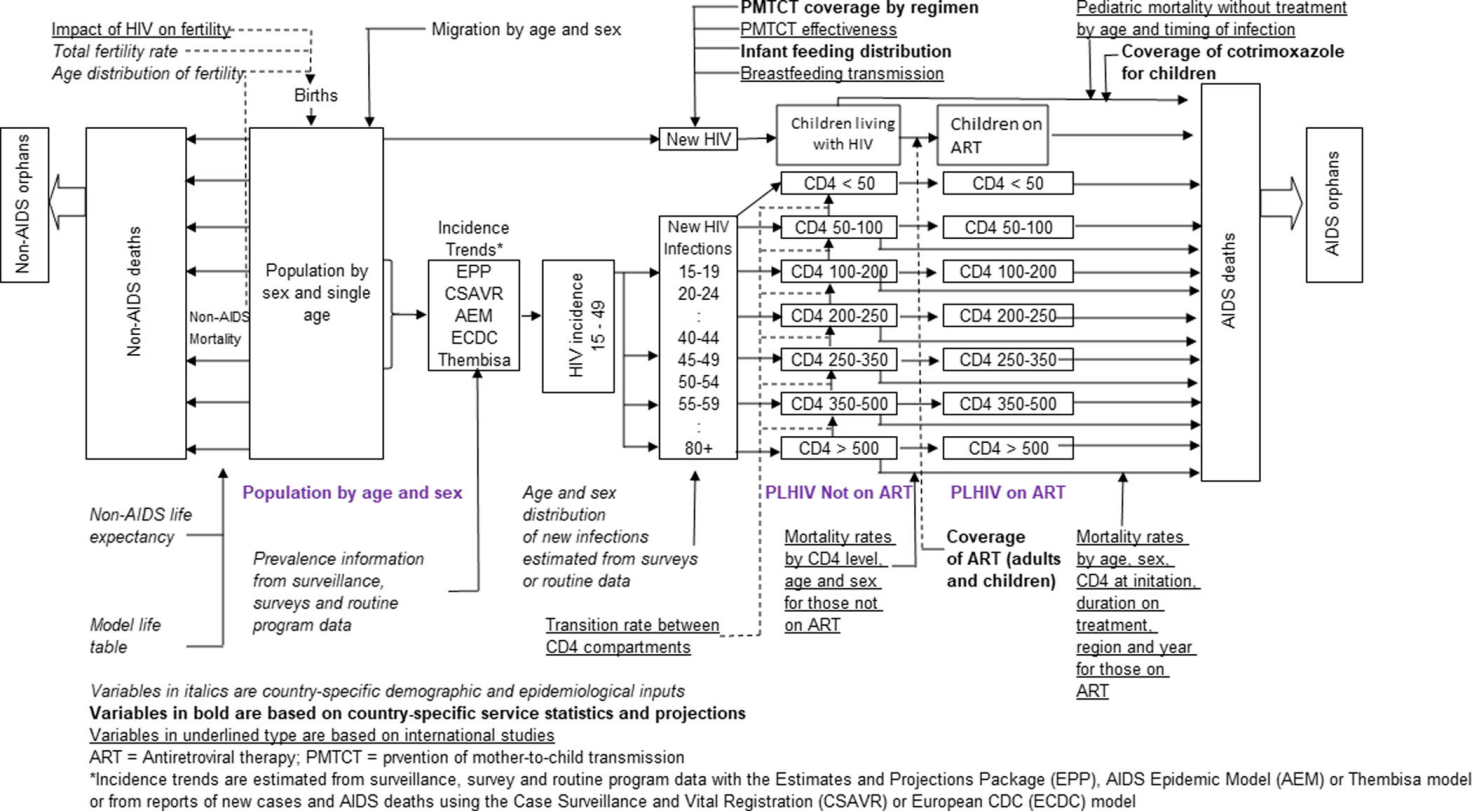
Structure of Spectrum/AIM model.

Most countries prepare annual estimates of key HIV indicators which are used for national planning and published annually by UNAIDS [1]. UNAIDS provides technical assistance through the provision of tools, training and assessment of data quality. The Spectrum/AIM model is one of the key tools used to produce the HIV estimates (Figure 1). In the model, each year the population ages and is subject to non‐AIDS mortality, migration and the risk of HIV infection. New child infections are determined from prevalence among pregnant women, use of antiretroviral drugs (ARVs) and transmission rates by ARV regimen. New adult infections are based on incidence trends as estimated by exogenous models (Estimation and Projection Package [2], Case Surveillance and Vital Registration [3], AIDS Epidemic Model [4]). New infections are distributed by age, sex and CD4 count category and tracked over time as individual's age, progress to lower CD4 categories, initiate antiretroviral therapy (ART) or die. Rates of progression to lower CD4 categories and death on and off ART are derived from fitting to survey, cohort and patient data. Details of the methods and assumptions are available in previous publications [5, 6]. Spectrum is freely available and can be downloaded from https://avenirhealth.org/software‐spectrum.php or used through a web‐based application (https://aim.spectrumweb.org/).

Spectrum is updated annually in response to new data, new program priorities and new issues. This article describes the updates to data and methods incorporated into Spectrum for the 2021 HIV estimates.

## 2 METHODS

An expert working group (UNAIDS Reference Group on Estimates, Modelling and Projections [http://www.epidem.org]) reviews evidence and makes recommendations for Spectrum updates. Some of the major changes for 2020 are discussed in other articles in this supplement. This article describes changes for three topics that use new data to update parameter values (mapping the transition of children with HIV from CD4% to CD4 count at age 5, the continuation of pregnant women on ART during breastfeeding and breastfeeding patterns among women living with HIV), one topic that responds to new program priorities (understanding the size of the previously treated population) and one new issue (COVID‐19).

### 2.1 Mapping the transition from CD4% to CD4 count for children with HIV

In Spectrum, when children with HIV turn 5 years old they transition from compartments defined by CD4% to compartments defined by CD4 counts, based on a matrix of transition probabilities. The probabilities were informed by data from the HIV Pediatric Prognostic Markers Collaborative Study (HPPMCS) [7, 8]. We used new data from the International epidemiological Databases to Evaluate AIDS (IeDEA) [9, 10] to update these transition probabilities.

The dataset (n=4851) includes children who initiated treatment during 2003 to 2017 within a year of their fifth birthday, and from five regions — two‐thirds of the children (65%) are from southern Africa, 18 and 8% from East and West Africa respectively, and 9 and 1% from Asia‐Pacific and the Caribbean, central and South America region, respectively. There are approximately equal numbers of males and females. Children had both CD4% and CD4 count values at ART initiation recorded, and the values were measured on the same date, closest to, and within 6 months before and 2 weeks after, the ART start date.

For each CD4% category, the relative frequency of CD4 count categories was measured using categories consistent with the Spectrum compartments.

### 2.2 ART continuation during breastfeeding

Program data on the number of women with HIV receiving ART while pregnant or breastfeeding are used to calculate the number of children who acquire HIV. Programs frequently report the number of women on ART at delivery, but generally do not include information about ART continuation during breastfeeding. The prior Spectrum default assumption, based on a 2018 literature review [5], was that 1.6% of women disengage from care and stop ART per month of breastfeeding. We undertook a literature review and meta‐analysis to update this assumption and account for the potential impact of silent transfers (i.e., unrecognized transfers of care between clinics) on estimates of ART continuation during breastfeeding.

We reviewed conference abstracts (CROI and IAS for 2017–2019) and the published literature (PubMed, EMBASE and Web of Science from 1 January 2012 to 30 September 2019) for studies reporting the proportion of women engaged in HIV care postpartum. We assumed that all women engaged in HIV care were on ART; breastfeeding practices were not reported in most studies, so we used engagement in care postpartum as a proxy for engagement in care during breastfeeding. Studies were excluded if they took place before the implementation of guidelines for lifelong ART for all pregnant women with HIV. We identified 1641 unique records that were possibly relevant and, after review, used 54 records for the analysis (Appendix S1). More than 80% of participants in the included studies were from sub‐Saharan Africa, with the rest from Haiti, Europe and the United States.

Maternal engagement in care was analysed by UNAIDS region at delivery and then at 1–6, 7–12 and 13–24 months postpartum among women who were in care at delivery (see Appendix S1 for full details regarding data extraction and analysis). At all time points, reported patient transfers and deaths were removed from the denominator of women at risk for disengagement. Three studies reported data on silent transfers among postpartum women identified via patient tracing and/or database review; in these, an average of 34% of participants originally classified as lost to follow‐up had transferred to another clinic and was still engaged in care [11, 12, 13]. Therefore, a 34% silent transfer rate was applied to the proportion of postpartum women reported as lost to follow‐up in studies that did not measure transfers. After incorporating this adjustment, we conducted a meta‐analysis to estimate the pooled proportion of women engaged in care at each time point postpartum. We then derived a constant rate of postpartum disengagement from care and translated this rate to a monthly risk. As disengagement was more frequent in the early postpartum period, we calculated monthly risks separately for the first 12 months postpartum and after 12 months postpartum.

### 2.3 Updates to breastfeeding patterns in sub‐Saharan Africa

Breastfeeding inputs to Spectrum quantify the proportion of women with HIV currently breastfeeding by lastborn child's age, maternal ART status and year. For previous estimates, most sub‐Saharan African countries used breastfeeding patterns estimated in household survey respondents regardless of HIV status, which may bias breastfeeding patterns needed for Spectrum if practices vary by HIV status.

We estimated breastfeeding patterns by region (central, eastern, southern and western Africa), HIV status, and calendar year using data from 64 nationally representative household surveys (Demographic and Health Surveys [14, 15], AIDS Indicator Surveys [16, 17] and Population‐based HIV Impact Assessment [PHIA] surveys [18, 19, 20]) from 31 countries (Appendix S2). We analysed surveys conducted in 2003‐2018 that included HIV testing and elicited current breastfeeding status from mothers who gave birth in the preceding 3 years. We did not model ARV status effects because these were only measured in the three PHIA surveys included. We excluded mothers whose lastborn child was not alive. The analysis dataset included 155,814 mothers, 9405 of whom tested HIV‐positive.

Breastfeeding patterns in Spectrum pertain to mothers with HIV only. However, we estimated breastfeeding patterns stratified by maternal HIV status as the relatively large numbers of HIV‐negative survey respondents allowed more robust identification of national and temporal patterns. We modelled proportions currently breastfeeding b by country, year t, child's age a and HIV status h

$$b\;\left(t,a,h\right)=\;u\left(t,h\right)F\left(a;m\left(t,h\right),s\left(t\right)\right)$$

as a function of the proportion u of mothers who breastfeed initially and their median duration of breastfeeding m. Breastfeeding proportions fall with child age according to the log‐logistic survival function F with shape s. We defined constituent terms as follows:

$$\begin{array}{ccc} \mathrm{logit}\left(u\left(t,h\right)\right) & = & \mathrm{logit}\left(\theta \right)+{\theta^{\prime }}_{r}t+\left\{\rho_{r}+{\rho^{\prime }}_{r}t\right\}\\ \ln\left(m\left(t,h\right)\right) & = & \mu +{\mu^{\prime }}_{r}t+\left\{\lambda_{r}+{\lambda^{\prime }}_{r}t\right\}\\ \ln\left(s\left(t\right)\right) & = & \sigma +{\sigma^{\prime }}_{r}t \end{array}$$

Terms in braces only apply to women with HIV. Free model parameters are country effects (θ, μ, σ) and regional coefficients for time ($\theta_{r}^{\prime }$, $\mu_{r}^{\prime }$, $\sigma_{r}^{\prime }$), HIV status ($\rho_{r}$, $\lambda_{r}$) and time‐HIV interactions ($\rho_{r}^{\prime }$, $\lambda_{r}^{\prime }$). We modeled temporal and HIV status effects regionally because there were generally too few surveys per country and HIV‐positive mothers per survey to support estimation of country‐level effects. We estimated temporal effects using 2010 as t=0. We used binomial likelihood to fit aggregate count data and estimated model parameters via maximum likelihood using Stan [21].

### 2.4 Previously treated populations

Efforts to increase ART coverage have usually focused on testing to identify people living with HIV (PLHIV) and linking them to care. As ART coverage and knowledge of status increase, a larger proportion of those not on ART may have previously been on treatment but disengaged from care. Different approaches may be needed to re‐engage them in care. This issue may be increasingly important if disruptions due to COVID‐19 increase disengagement as discussed in the next section.

**Figure 2:**
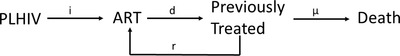
Treatment dynamics.

To understand these dynamics, we have added to Spectrum an estimate of the size of the previously treated population. PLHIV initiate ART at rate *i*. Those on ART may die from HIV or non‐HIV causes (not shown in the figure) or disengage from treatment at rate *d*. Those who were previously treated may remain in that category, die (at rate *μ*) or re‐engage at rate *r* (Figure 2).

Program records indicate the number of people on ART over time. Program data often report rates of loss to follow‐up, but this is not the same as disengagement since some people who are lost to follow‐up at a particular clinic may have transferred to another clinic or died. Similarly, little information is available on rates of re‐engagement in care. Spectrum calculates the mortality of PLHIV not on ART, but we do not know if those who have been previously treated have the same mortality rates or likelihood of enrolling in ART as those who are treatment naïve. Therefore, displaying the previously treated population in Spectrum allows for these rates to be varied by the user to test the sensitivity of the conclusions to those assumptions.

### 2.5 Accounting for the impact of COVID‐19 in 2020

The COVID‐19 pandemic has disrupted health services in many places. Some services have been unavailable for certain periods of time and some people have avoided visiting health facilities for fear of COVID‐19. Some countries have reported dips in the number of people receiving ART for 1 or 2 months and many have reported reductions in the number of people newly starting ART of up to 50% or more [22]. In some countries, numbers have rebounded to January 2020 levels whereas in others they are still depressed. Although Spectrum calculations are done in one‐tenth‐year time steps, data on the number of people on ART are typically entered annually as of December 31st each year. Spectrum uses linear interpolation between the December 31st figures to compute the intermediate time points. This approach could over‐state the effects of ART on mortality if declines in ART services during 2020 are masked by a rebound by December 31st. Therefore, Spectrum has been modified to accept monthly figures for the number of patients on ART.

PLHIV not on ART are tracked in Spectrum by CD4 category. Those on ART are characterized by CD4 count at the initiation. Those who disengaged from ART were assigned to the CD4 category they were in at treatment initiation. This might over‐estimate mortality by ignoring the increase in CD4 counts while on treatment unless CD4 counts drop rapidly back to pre‐treatment levels.

**Figure 3:**
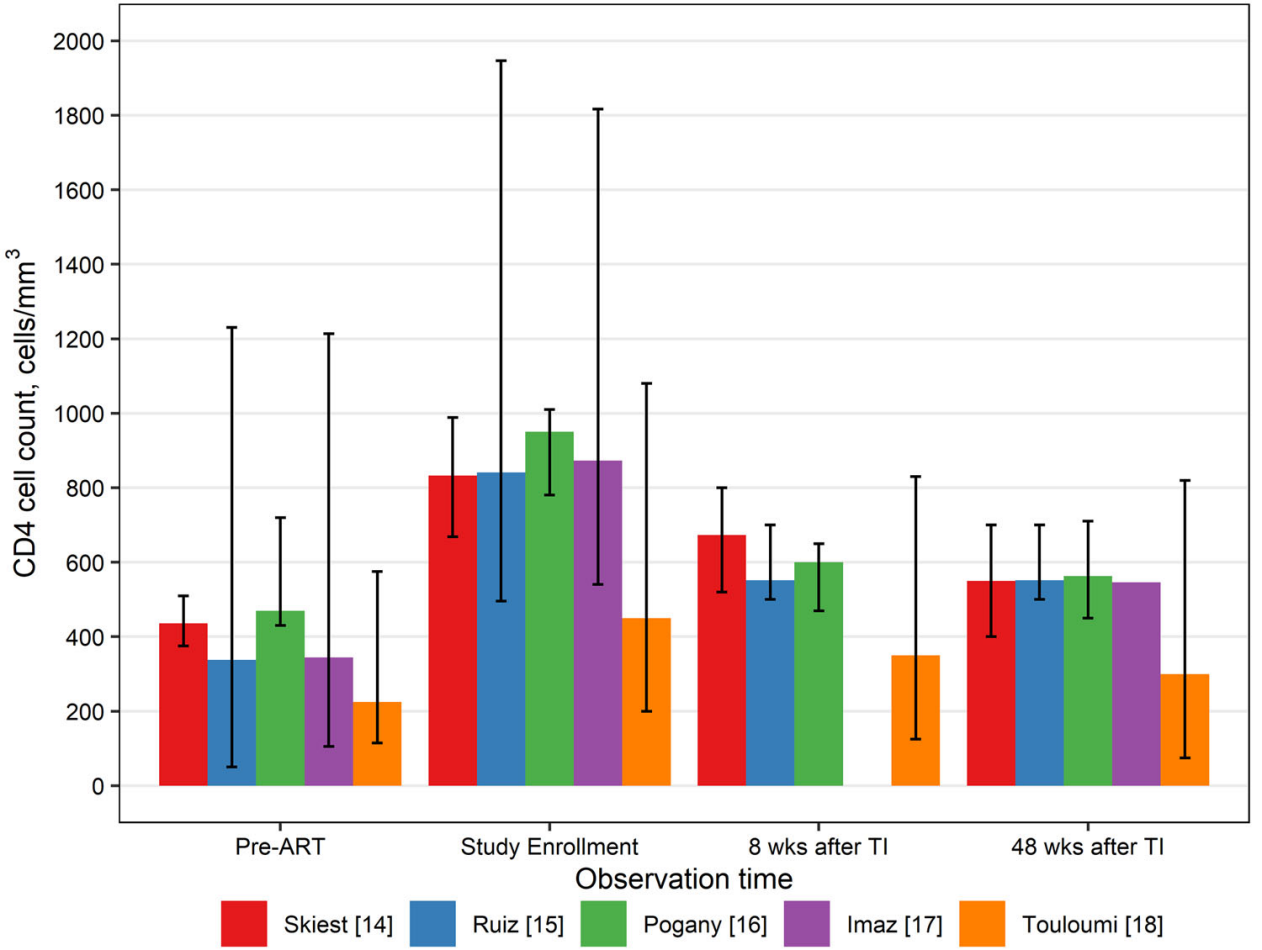
CD4 counts of patient's pre‐ART, on ART and after treatment interruption (TI).

Several studies of treatment interruption report patient CD4 counts before, during and after treatment interruption [23, 24, 25, 26, 27]. Figure 3 shows data from five studies illustrating the increase in CD4 counts from pre‐ART to study enrolment (when patients had been on ART for some time) and the decline after treatment interruption. In four of the studies, patients with high and stable CD4 counts were selected for structured treatment interruption. Only the Touloumi study followed patients who dropped out of treatment. Eight weeks after treatment interruption 40 to 73% of the gains in CD4 counts while on treatment were lost and 57 to 80% were lost after 48 weeks. To replicate this dynamic, Spectrum now assigns people who disengage from care after being on treatment for at least 1 year to the CD4 category, one higher than they were in when they initiated treatment. For most applications, this produces an effect similar to that shown in Figure 3.

### 2.6 Ethics approval

Unless otherwise stated, data used for the analyses in this article were publicly available aggregate data for which consent is not required.

## 3 RESULTS

### 3.1 Mapping the transition from CD4% to CD4 count for children living with HIV

The IeDEA collaboration consolidates, curates and analyses existing clinical and epidemiological data on people living with HIV in routine‐care settings globally [9, 10]. Each treatment programme participating in IeDEA has obtained ethical approval from the appropriate local institutions to collect and share patient data, and each regional data centre has obtained ethical approval to analyse the consolidated, de‐identified data. All other data used for the analyses in this article were publicly available aggregate data for which consent is not required.

The transition probabilities resulting from our analysis of IeDEA data are shown in Table 1. Uncertainty ranges and the previous transition values are in Appendix S3.

**Table 1:** The relationship between CD4% and CD4 count at ART start, amongst children who initiate ART within a year of their fifth birthday

		Percent in CD4 count category (cells/mm^3^)^a^					
CD4 %^a^	n	[0‐199]	[200‐349]	[350‐499]	[500‐749]	[750‐1000]	[>1000]
[0‐4]	695	93.38	5.04	0.72	0.58	0.14	0.14
[5‐10]	1002	33.93	36.93	16.67	9.78	1.80	0.90
[11‐15]	1174	7.75	25.38	25.98	27.85	9.20	3.83
[16‐20]	819	3.17	14.65	24.66	31.26	16.85	9.40
[21‐25]	482	1.45	5.81	10.58	36.51	25.73	19.92
[26‐30]	305	1.31	3.61	9.51	29.51	22.3	33.77
[>30)	374	2.41	1.87	5.61	10.7	18.72	60.7
Total	4851	23.21	17.91	16.08	20.43	10.86	11.5

^a^ Numbers in brackets represent the bounds of each category.

For all countries in sub‐Saharan Africa, where the majority of children with HIV are located, the new transition probabilities result in an increase of 0.5% children living with HIV in 2019. For AIDS‐related deaths, the difference is larger. When applying the new transition probabilities retrospectively, in the early part of the epidemic there are fewer AIDS‐related deaths with the new probabilities, but after 2003, when ART starts to scale‐up, there are more deaths. Cumulative deaths from 1970 to 2019 are 1.8% lower, but estimated deaths in 2019 are 6% higher with the new transition probabilities.

### 3.2 ART continuation during breastfeeding

Pooled estimates of postpartum engagement in care are shown in Table 2. While there is variation by region, the 95% confidence bounds are wide. Therefore, we use the overall average for all countries, which is 1.2% per month disengagement from care during the first 12 months and 0.7% per month from the 13th month to the end of breastfeeding.

**Table 2:** Summary of continuation on ART and disengagement from care during breastfeeding

	Women with HIV engaged in care and on ART at each time point, % (95% CI)^a^				Monthly risk of disengagement from care, % (95% CI)	
Region	Delivery^b^	1‐6 months postpartum	7‐12 months postpartum	13‐24 months postpartum	1‐12 months postpartum	13+ months postpartum
West and central Europe and North America	N/A	63 (43, 82)	88 (70, 100)	55 (28, 81)	1.1	3.3
Eastern Africa	73 (54, 91)	83 (79, 88)	81 (74, 86)	81 (61, 98)	1.8	−0.1
Southern Africa	79 (67, 90)	88 (82, 93)	87 (79, 94)	79 (70, 88)	1.1	0.7
Latin America and the Caribbean	75 (73, 76)	82 (80, 85)	78 (65, 90)	N/A	2.1	N/A
West and central Africa	86 (82, 91)	86 (77, 94)	85 (82, 88)	82 (77, 88)	1.3	0.2
Overall	78 (70, 86)	87 (80, 93)	87 (81, 92)	79 (66, 90)	1.2	0.7

ART, antiretroviral therapy; CI, confidence interval.

N/A: Data not available.

^a^ Proportions of women engaged in care at each postpartum time point are among women who were engaged in care at delivery.

^b^ The silent transfer adjustment was not applied to estimates of engagement in care at delivery.

The new recommended values are lower than the previous values and, therefore, will result in lower estimates of new child infections. For the 2020 round of estimates, 86% of countries used the default values for ART continuation with the rest using their own estimates. If all countries using the recommended values updated to the new values then the estimated number of new child infections in 2019 would be 0.9% lower (1300 fewer new infections).

### 3.3 Updates to breastfeeding patterns in sub‐Saharan Africa

**Figure 4:**
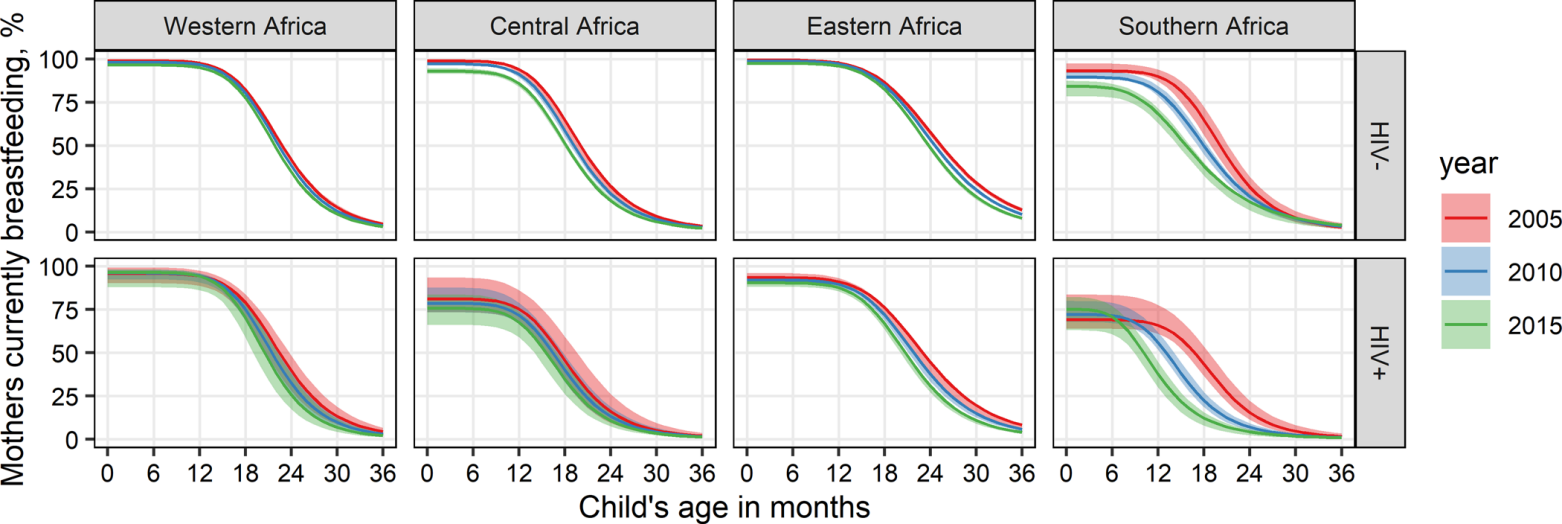
**Modelled breastfeeding duration by region and HIV status in 2005, 2010 and 2015**. Regional trends shown are based on average country effects. Maximum likelihood point estimates (solid curves) and 95% central credible intervals (shaded areas) are shown.

We found that women with HIV were less likely to breastfeed initially and had a shorter breastfeeding duration compared to HIV‐negative mothers (Figure 4). Breastfeeding patterns varied substantially by region and durations were shortest in southern Africa. Breastfeeding durations fell over time, with the largest changes apparent in southern Africa. These patterns are included in Spectrum using country‐specific values for initial breastfeeding, median duration and shape parameters in countries with household surveys, or regional averages in countries without.

These changes result in fewer new paediatric HIV infections throughout the sub‐Saharan African epidemic. At their peak in 2000, new paediatric HIV infections estimates were 2.6% lower with proposed (430,200) compared to original (441,500) inputs, while estimates in 2017 were 10.7% lower (proposed: 166,900; original: 186,900). More information in Appendix S2.

### 3.4 Previously treated populations

**Figure 5:**
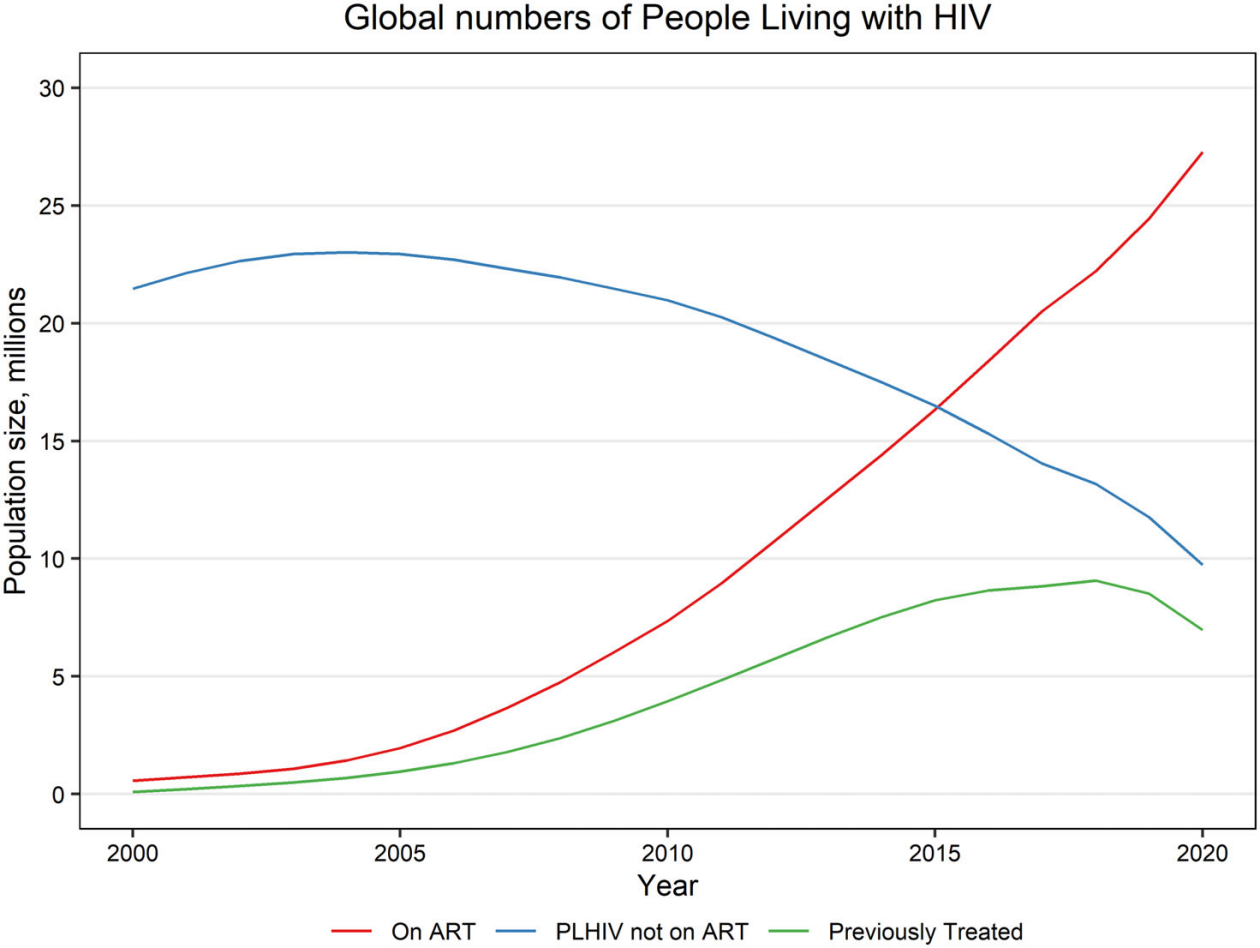
Numbers of people living with HIV by ART status. Based on reported numbers of people on ART by year and assumed annual retention on ART of 83% and the same rates of mortality and ART initiation for those previously on ART and never on ART. Note that the line for PLHIV not on ART includes those previously treated. ART, antiretroviral therapy; PLHIV, people living with HIV.

In countries with high ART coverage, it is likely that a large portion of PLHIV not on treatment have been previously treated, even when disengagement from treatment is low. These dynamics are illustrated for the world in Figure 5 assuming 83% annual retention on ART and the same mortality and probability of initiating ART among those previously treated as among those never on treatment. With those assumptions, 72% of PLHIV not on ART would be previously treated. Variations in mortality of those previously treated have little effect. However, the percentage previously treated drops to 43% if those previously treated are 50% more likely to re‐initiate treatment than those who are treatment naïve or if annual retention increases to 94%.

### 3.5 Accounting for the impact of COVID‐19 in 2020

The changes in CD4 categories for those who disengage from care will affect country projections differently depending on the level of disengagement as well as the recent scale‐up pattern of ART. Seventy‐five percent of countries do not enter information on disengagement so there will be no impact of this change. Among the remaining countries, annual disengagement is 1‐7% in most countries with only 10 countries reporting higher rates. For countries reporting disengagement, the effect of the change is to reduce the estimated number of cumulative AIDS deaths over the past 20 years by 0‐3% depending on the level of disengagement and the pattern of ART scale‐up. Country estimates that include the effects of COVID‐19 will become available later in 2021.

## 4 DISCUSSION

The changes described in this article are intended to address new issues, incorporate new data, include information from newly published studies and benefit from new analyses. Except for the service disruptions due to COVID‐19, the other updates all use data from several countries to recommend default values to be used when country‐specific information is lacking.

There were several limitations in these data used. For breastfeeding continuation, there was substantial heterogeneity in the reported definitions of engagement in care, insufficient data regarding engagement in care after patient transfers, scarce data on postpartum re‐engagement in care and lack of data specific to breastfeeding populations. Additional data on postpartum engagement in care among breastfeeding women, particularly in settings outside of eastern and southern Africa, are needed to further refine these estimates.

Most changes to methods or assumptions in Spectrum will influence not only the new 2020 estimates but also estimates for prior years. For example, a change to the assumption of ART continuation during breastfeeding will affect the estimates of mother‐to‐child transmission since the scale‐up of ART began. Also new survey‐based information on behaviours, such as breastfeeding, usually refers to a period several years ago. Changes to past estimates can cause problems for national programs. For example, there is a natural tendency to compare the new 2020 estimate with the 2019 estimate made during the previous year as if it represented a trend. To guard against this, new estimates always contain complete time histories of all indicators. If estimates change substantially, there can be program implications (for example, ART coverage may be lower or higher than previously estimated) as well as implications for meeting promised targets such as Global Fund performance targets. For these reasons, updates are only implemented when the Reference Group determines that they represent a clear improvement of previous approaches. Every effort is made through training, briefs and publications, such as this one, to communicate the reasons for the changes and to make sure the effects can be understood and explained. Country analysts are free to change any of the global parameter values in the model but are encouraged to do so only if they can demonstrate that they have good data to justify the change.

## 5 CONCLUSIONS

The updated Spectrum/AIM model uses newly available data and studies to improve the estimation of paediatric and adult HIV indicators. Improved estimates will support efforts to effectively address the HIV/AIDS pandemic and evaluate progress towards global goals of eliminating AIDS as a public health threat by 2030.

## FUNDING

Work by J.S. and R.G. was funded by a Grant from the Bill and Melinda Gates Foundation (OPP 1191665). Work by C.M.D. was funded by the *Eunice Kennedy Shriver* National Institute of Child Health and Human Development (K08HD101342). The IeDEA regions are supported by the National Institute on Drug Abuse (NIDA); the National Heart, Lung, and Blood Institute (NHLBI); the National Institute on Alcohol Abuse and Alcoholism (NIAAA); the National Institute of Diabetes and Digestive and Kidney Diseases (NIDDK); the Fogarty International Center (FIC); the Eunice Kennedy Shriver National Institute of Child Health and Human Development (NICHD); the National Cancer Institute (NCI); the National Institute of Allergy and Infectious Diseases (NIAID); the National Institute of Mental Health (NIMH); the Office of the Director, National Institutes of Health (OD) and the National Library of Medicine (NLM). The IeDEA regions are supported by grants U01AI069907 (TREAT Asia Pediatric HIV Observational Database), U01AI069923 (Caribbean, Central and South America network for HIV epidemiology), U01AI069911 (East Africa), U01AI069924 (Southern Africa) and U01AI069919 (West Africa). Informatics resources are supported by the Harmonist project (R24AI124872).

## DISCLAIMER

The content is solely the responsibility of the authors and does not necessarily represent the official views of the National Institutes of Health. Site investigators and cohorts are listed in the provided Funding section.

## COMPETING INTERESTS

The authors declare that they have no conflict of interests.

## AUTHORS’ CONTRIBUTIONS

JS coordinated the manuscript preparation, wrote the introduction and conclusions, and did the analysis and wrote the sections on previously treated populations and modifications for COVID‐19. RK conducted the analysis and wrote the section on mapping paediatric CD4 categories. CMD did the analysis and wrote the section on ART continuation during breastfeeding. RG did the analysis and wrote the section on breastfeeding patterns.

## Supporting information

**Appendix S1**. Updates to the Spectrum/AIM Model continuation on ART during breastfeeding

**Appendix S2**. Updates to breastfeeding patterns in sub‐Saharan Africa for the 2020 round of HIV estimates

**Appendix S3**. Mapping the transition from CD4 percent to CD4 count for HIV‐infected children
