# Supplementary material for: Updates to the Spectrum/AIM model for the UNAIDS 2020 HIV estimates
Source: J Int AIDS Soc. 2021 Sep 21;24(Suppl 5):e25778. doi: 10.1002/jia2.25778 (PMC8454674; doi:10.1002/jia2.25778)
Supplement: Supplementary file 1 — Appendix S1. Updates to the Spectrum/AIM Model continuation on ART during breastfeeding [file JIA2-24-e25778-s001.docx]

**Supplementary Appendix 1: Updates to the Spectrum/AIM Model continuation on ART during breastfeeding**

Literature search

On September 30, 2019, we conducted a search of conference abstracts (CROI and IAS from for 2017-2019) and the PubMed, EMBASE, and Web of Science databases for articles published in English, French, or Spanish between January 1, 2012 and September 30, 2019 using terms related to HIV, pregnancy, breastfeeding, and engagement in care, as specified in Supplemental Table 1. Included studies reported proportions of women who were engaged in HIV care at one or more timepoints postpartum among all women in the starting cohort; these studies did not necessarily specify whether or not women were breastfeeding at each timepoint. Studies were excluded if they were conducted prior to implementation of guidelines for lifelong ART for pregnant and breastfeeding women with HIV, or if they were limited to a particular patient sub-population (e.g., female sex workers or adolescents). If multiple studies were identified that presented data on the same, or potentially overlapping, cohorts of women, only the study that provided data on the greatest number of subjects was included in the analysis.

The search identified 1641 unique records, of which 1461 were deemed irrelevant to the study question based on title and abstract screening (Supplemental Figure 1). The remaining 180 full text records were screened; 129 of these records were excluded due to reporting on study populations that predated lifelong ART guidelines, missing data on the proportion of women engaged in care postpartum, study design, and including cohorts that overlapped with other studies included in the analysis. Bibliography review of the 180 screened full texts identified 3 additional records that met inclusion criteria. Therefore, a total of 54 records contributed data towards the meta-analysis of postpartum engagement in HIV care [1–54]. One additional article identified in the full text review contributed data on silent transfers, but did not provide data towards the meta-analysis of postpartum engagement in HIV care [55].

Data extraction and analysis

We extracted data on the proportion of women in the starting cohort who were engaged in HIV care at delivery, 1-6 months, 7-12 months, and 13-24 months postpartum, by UNAIDS region [56]. Given the relative abundance of data from the Eastern and Southern African region, this region was further subdivided into Southern Africa (eSwatini, Malawi, Mozambique, South Africa, Zambia, and Zimbabwe) and Eastern Africa (Ethiopia, Kenya, Rwanda, Tanzania, and Uganda). Some studies reported engagement in care among pregnant or breastfeeding women at a certain month after ART start rather than at a discrete timepoint postpartum. For these studies, if a median gestational age at antenatal care enrollment was specified, it was used to approximate time of ART start relative to delivery. For example, if median gestational age at antenatal enrollment was reported to be 28 weeks, this was assumed to be 3 months prior to delivery (40 weeks at term – 28 weeks gestational age = 12 weeks = 3 months). If that same study reported engagement in care at 12 months after ART start, this timepoint was interpreted as being the same as 9 months postpartum. If gestational age at antenatal care enrollment was not specified, we made the assumption that women who were ART naïve prior to pregnancy enrolled in antenatal care and started ART 5 months prior to delivery.

When data were available from both the intervention and control arms of a study, we only used the data from the control arm. If the study reported patient transfers and/or deaths, these individuals were removed from the denominator of women at risk for disengagement from care for calculations of proportions of women engaged in care at each timepoint postpartum. There were three studies identified in the search that reported data on silent transfers (i.e. unrecognized transfers of care between clinics that may result in some individuals being misclassified as lost to follow-up) among postpartum women with HIV [40,53,55]. In these studies, an average of 34% of postpartum women originally been classified as lost to follow-up had actually transferred to another clinic and were still engaged in care. Therefore, in studies in which transfers were not otherwise explicitly reported, we applied a “silent transfer adjustment,” by reclassifying 34% of postpartum individuals reported to be lost to follow-up as instead still engaged in care. This adjustment was not applied to estimates of engagement in care at delivery, as data on silent transfers for this timepoint were lacking.

We performed a meta-analysis to estimate the pooled proportion of women engaged in HIV care at delivery and each timepoint postpartum using the inverse variance heterogeneity model in MetaXL v5.3 [57,58]. When data were available, pooled proportions were presented separately by UNAIDS region, with Eastern and Southern Africa each also presented separately (Supplemental Figures 2-5). Using the pooled proportions of engagement in care at the 7-12 month and 13-24 month intervals, we derived a constant rate of postpartum disengagement from care (using the distal bound of each interval) and converted this rate to a monthly risk. In the majority of included studies, disengagement was more frequent in the early postpartum period, so we calculated monthly risks separately for the first 12 months postpartum and after 12 months postpartum (Table 2).

**Supplemental Table 1. PubMed^†^ search for literature review of continuation of ART during breastfeeding.**

|  | "HIV Infections"[Mesh] OR “Human immunodeficiency virus”[tiab] OR HIV[tiab] OR “AIDS”[tiab] |
| --- | --- |
| **AND** | "Pregnancy"[Mesh] OR "Pregnant Women"[Mesh] OR "Prenatal Care"[Mesh] OR "Perinatal Care"[Mesh] OR "Breast Feeding"[Mesh] OR "Postnatal Care"[Mesh] OR "Postpartum Period"[Mesh] OR pregnant[tiab] OR “pregnancy”[tiab] OR antenatal[tiab] OR antepartum[tiab] OR prenatal[tiab] OR “pre natal”[tiab] OR perinatal[tiab] OR puerperium[tiab] OR postnatal[tiab] OR “post natal”[tiab] OR postpartum[tiab] OR “post partum”[tiab] OR breastfeeding[tiab] OR “breast feeding”[tiab] |
| **AND** | "Retention in Care"[Mesh] OR "Lost to Follow-Up"[Mesh] OR retention[tiab] OR retain*[tiab] OR LTFU[tiab] OR “lost to follow up”[tiab] OR “loss to follow up”[tiab] OR default[tiab] OR attrition[tiab] OR engagement[tiab] OR engaged[tiab] OR attendance[tiab] OR disengagement[tiab] OR disengaged[tiab] OR disengage[tiab] OR “drop out”[tiab] |
| **NOT** | Qualitative[tiab] OR interviews[tiab] OR “focus group*”[tiab] OR “case report”[tiab] OR “pre-exposure prophylaxis”[tiab] |

^†^ Search terms were adapted to equivalent formats for EMBASE and Web of Science.

**Supplemental Figure 1. PRISMA diagram of literature search for continuation of ART during breastfeeding.**


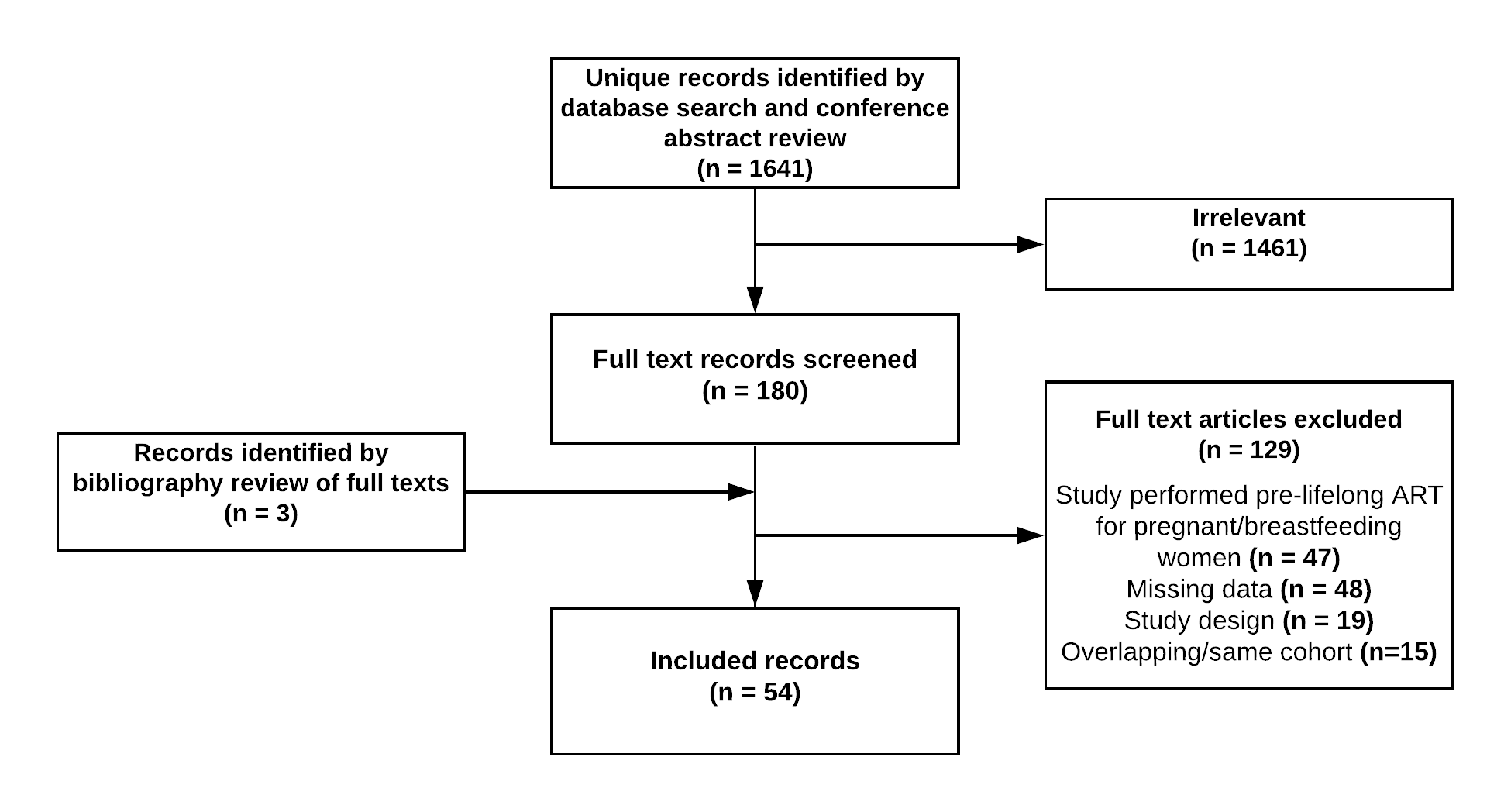


**Supplemental Figure 2. Forest plot of engagement in HIV care at delivery, by region.**

**Supplemental Figure 3. Forest plot of engagement in HIV care at 1-6 months postpartum, by region.**

**Supplemental Figure 4. Forest plot of engagement in HIV care at 7-12 months postpartum, by region.**

**Supplemental Figure 5. Forest plot of engagement in HIV care at 13-24 months postpartum, by region.**

**REFERENCES**

1. Abrams EJ, Langwenya N, Gachuhi A, Zerbe A, Nuwagaba-Biribonwoha H, Mthethwa-Hleta S, et al. Impact of universal antiretroviral therapy for pregnant and postpartum women on antiretroviral therapy uptake and retention. AIDS (London, England). 2019;33(1):45–54.

2. Adams JW, Brady KA, Michael YL, Yehia BR, Momplaisir FM. Postpartum Engagement in HIV Care: An Important Predictor of Long-term Retention in Care and Viral Suppression. Clin Infect Dis. 2015 Dec 15;61:1880–7.

3. Adhikari EH, Yule CS, Roberts SW, Rogers VL, Sheffield JS, Kelly MA, et al. Factors Associated with Postpartum Loss to Follow-Up and Detectable Viremia After Delivery Among Pregnant Women Living with HIV. AIDS patient care and STDs. 2019;33(1):14–20.

4. Akama E, Nimz A, Blat C, Moghadassi M, Oyaro P, Maloba M, et al. Retention and viral suppression of newly diagnosed and known HIV positive pregnant women on Option B+ in Western Kenya. AIDS Care. 2019 Mar;31(3):333–9.

5. Alhaj M, Amberbir A, Singogo E, Banda V, van Lettow M, Matengeni A, et al. Retention on antiretroviral therapy during Universal Test and Treat implementation in Zomba district, Malawi: a retrospective cohort study. Journal of the International AIDS Society. 2019;22(2):e25239.

6. Atanga PN, Ndetan HT, Achidi EA, Meriki HD, Hoelscher M, Kroidl A. Retention in care and reasons for discontinuation of lifelong antiretroviral therapy in a cohort of Cameroonian pregnant and breastfeeding HIV-positive women initiating ‘Option B+’ in the South West Region. Trop Med Int Health. 2017 Feb;22:161–70.

7. Chan AK, Kanike E, Bedell R, Mayuni I, Manyera R, Mlotha W, et al. Same day HIV diagnosis and antiretroviral therapy initiation affects retention in Option B+ prevention of mother-to-child transmission services at antenatal care in Zomba District, Malawi. J Int AIDS Soc. 2016;19:20672.

8. Chimbwandira F, Mhango E, Makombe S, Midiani D, Mwansambo C, Njala J, et al. Impact of an Innovative Approach to Prevent Mother-to-Child Transmission of HIV - Malawi, July 2011-September 2012. Mmwr-Morbidity and Mortality Weekly Report. 2013;62(8):148–51.

9. Cichowitz C, Mazuguni F, Minja L, Njau P, Antelman G, Ngocho J, et al. Vulnerable at Each Step in the PMTCT Care Cascade: High Loss to Follow Up During Pregnancy and the Postpartum Period in Tanzania. AIDS and behavior. 2019;23(7):1824–32.

10. Decker S, Rempis E, Schnack A, Braun V, Rubaihayo J, Busingye P, et al. Prevention of mother-to-child transmission of HIV: Postpartum adherence to Option B+ until 18 months in Western Uganda. PloS one. 2017;12(6).

11. Deschamps MM, Jannat-Khah D, Rouzier V, Bonhomme J, Pierrot J, Lee MH, et al. Fifteen years of HIV and syphilis outcomes among a prevention of mother-to-child transmission program in Haiti: from monotherapy to Option B. Tropical medicine & international health : TM & IH. 2018;23(7):724–37.

12. Dzangare J, Takarinda KC, Harries AD, Tayler-Smith K, Mhangara M, Apollo TM, et al. HIV testing uptake and retention in care of HIV-infected pregnant and breastfeeding women initiated on ‘Option B+’ in rural Zimbabwe. Trop Med Int Health. 2016 Feb;21:202–9.

13. Erlwanger AS, Joseph J, Gotora T, Muzunze B, Orne-Gliemann J, Mukungunugwa S, et al. Patterns of HIV care clinic attendance and adherence to antiretroviral therapy among pregnant and breastfeeding women living with HIV in the context of option b+ in Zimbabwe. Journal of Acquired Immune Deficiency Syndromes. 2017;75((Farley T.) Sigma3 Services, Nyon, Switzerland):S198–206.

14. Etoori D, Kerschberger B, Staderini N, Ndlangamandla M, Nhlabatsi B, Jobanputra K, et al. Challenges and successes in the implementation of option B+ to prevent mother-to-child transmission of HIV in southern Swaziland. BMC Public Health. 2018 Mar 20;18(1):374.

15. Ford D, Muzambi M, Nkhata MJ, Abongomera G, Joseph S, Ndlovu M, et al. Implementation of Antiretroviral Therapy for Life in Pregnant/Breastfeeding HIV+ Women (Option B+) Alongside Rollout and Changing Guidelines for ART Initiation in Rural Zimbabwe: The Lablite Project Experience. Journal of acquired immune deficiency syndromes (1999). 2017;74(5):508–16.

16. Foster G, Orne-Gliemann J, Font H, Kangwende A, Magezi V, Sengai T, et al. Impact of Facility-Based Mother Support Groups on Retention in Care and PMTCT Outcomes in Rural Zimbabwe: The EPAZ Cluster-Randomized Controlled Trial. J Acquir Immune Defic Syndr. 2017 Jun 1;75 Suppl 2:S207–15.

17. Gamell A, Luwanda LB, Kalinjuma AV, Samson L, Ntamatungiro AJ, Weisser M, et al. Prevention of mother-to-child transmission of HIV Option B+ cascade in rural Tanzania: The One Stop Clinic model. PLoS One. 2017;12:e0181096.

18. Guillaine N, Mwizerwa W, Odhiambo J, Hedt-Gauthier BL, Hirschhorn LR, Mugwaneza P, et al. A Novel Combined Mother-Infant Clinic to Optimize Post-Partum Maternal Retention, Service Utilization, and Linkage to Services in HIV Care in Rural Rwanda. Int J MCH AIDS. 2017;6:36–45.

19. Haas AD, Tenthani L, Msukwa MT, Tal K, Jahn A, Gadabu OJ, et al. Retention in care during the first 3 years of antiretroviral therapy for women in Malawi’s option B+ programme: an observational cohort study. Lancet HIV. 2016 Apr;3:e175-82.

20. Harrington BJ, DiPrete BL, Jumbe AN, Ngongondo M, Limarzi L, Wallie SD, et al. Safety and efficacy of Option B+ ART in Malawi: few severe maternal toxicity events or infant HIV infections among pregnant women initiating tenofovir/lamivudine/efavirenz. Trop Med Int Health. 2019 Oct;24(10):1221–8.

21. Hauser BM, Miller WC, Tweya H, Speight C, Mtande T, Phiri S, et al. Assessing Option B+ retention and infant follow-up in Lilongwe, Malawi. Int J STD AIDS. 2017 Jan 1;62:956462417721658.

22. Hosseinipour M, Nelson JAE, Trapence C, Rutstein SE, Kasende F, Kayoyo V, et al. Viral Suppression and HIV Drug Resistance at 6 Months Among Women in Malawi’s Option B+ Program: Results From the PURE Malawi Study. J Acquir Immune Defic Syndr. 2017 Jun 1;75 Suppl 2:S149–55.

23. Jones D, Rodriguez V, Weiss S, Peltze K. Challenges to engagement and retention of HIV-infected perinatal women in rural South Africa. International Journal of Behavioral Medicine. 2018;25:S108–9.

24. Joseph J, Suggu K, Hariharan N, Esiru G, Yavuz E, Gross J, et al. Increasing retention of HIV-positive pregnant and postnatal women and HIV-exposed infants: Measuring the effects of follow-up activities and improved patient management in rural Uganda. Journal of the International AIDS Society. 2016;19((Mirembe B.) Clinton Health Access Initiative, Kampala, Uganda):25–6.

25. Kalua T, Tippett Barr BA, van Oosterhout JJ, Mbori-Ngacha D, Schouten EJ, Gupta S, et al. Lessons Learned From Option B+ in the Evolution Toward ‘Test and Start’ From Malawi, Cameroon, and the United Republic of Tanzania. J Acquir Immune Defic Syndr. 2017 May 1;75 Suppl 1:S43–50.

26. Kamuyango AA, Hirschhorn LR, Wang W, Jansen P, Hoffman RM. One-year outcomes of women started on antiretroviral therapy during pregnancy before and after the implementation of Option B+ in Malawi: A retrospective chart review. World J AIDS. 2014 Sep 1;4(3):332–7.

27. Karajeanes E, Bila D, Augusto O, Lain M, Muianga B, Muluana C, et al. Uptake and retention in care of pregnant women starting option B+ in maputo. Topics in Antiviral Medicine. 2017;25(1):326s.

28. Kasonde P, Sitenge G, Mwale C, Malebe T, Kabaso M, Welsh M. Implementation of Option B+ in selected parts of northern Zambia: Early lessons learnt. AIDS Research and Human Retroviruses. 2016;32((Kasonde P.; Sitenge G.; Mwale C.; Malebe T.; Kabaso M.; Welsh M.) FHI 360 Zambia, Zambia):196.

29. Kim MH, Ahmed S, Hosseinipour MC, Giordano TP, Chiao EY, Yu X, et al. The Impact of Option B plus on the Antenatal PMTCT Cascade in Lilongwe, Malawi. Jaids-Journal of Acquired Immune Deficiency Syndromes. 2015;68(5):E77–83.

30. Kiwanuka G, Kiwanuka N, Muneza F, Nabirye J, Oporia F, Odikro MA, et al. Retention of HIV infected pregnant and breastfeeding women on option B+ in Gomba District, Uganda: a retrospective cohort study. BMC Infect Dis. 2018 Oct 24;18(1):533.

31. Koole O, Houben RM, Mzembe T, Van Boeckel TP, Kayange M, Jahn A, et al. Improved retention of patients starting antiretroviral treatment in Karonga District, northern Malawi, 2005-2012. J Acquir Immune Defic Syndr. 2014 Sep 1;67:e27–33.

32. Koss CA, Natureeba P, Kwarisiima D, Ogena M, Clark TD, Olwoch P, et al. Viral Suppression and Retention in Care up to 5 Years After Initiation of Lifelong ART During Pregnancy (Option B+) in Rural Uganda. J Acquir Immune Defic Syndr. 2017 Mar 1;74:279–84.

33. Llenas-Garcia J, Wikman-Jorgensen P, Hobbins M, Mussa MA, Ehmer J, Keiser O, et al. Retention in care of HIV-infected pregnant and lactating women starting ART under Option B+ in rural Mozambique. Trop Med Int Health. 2016 Aug;21:1003–12.

34. Meade CM, Badell M, Hackett S, Mehta CC, Haddad LB, Camacho-Gonzalez A, et al. HIV Care Continuum among Postpartum Women Living with HIV in Atlanta. Infectious diseases in obstetrics and gynecology. 2019;2019:8161495.

35. Miller K, Muyindike W, Matthews LT, Kanyesigye M, Siedner MJ. Program Implementation of Option B+ at a President’s Emergency Plan for AIDS Relief-Supported HIV Clinic Improves Clinical Indicators But Not Retention in Care in Mbarara, Uganda. AIDS Patient Care STDS. 2017 Aug;31:335–41.

36. Mitiku I, Arefayne M, Mesfin Y, Gizaw M. Factors associated with loss to follow-up among women in Option B+ PMTCT programme in northeast Ethiopia: a retrospective cohort study. J Int AIDS Soc. 2016;19:20662.

37. Muhumuza S, Akello E, Kyomugisha-Nuwagaba C, Baryamutuma R, Sebuliba I, Lutalo IM, et al. Retention in care among HIV-infected pregnant and breastfeeding women on lifelong antiretroviral therapy in Uganda: A retrospective cohort study. PloS one. 2017;12(12):e0187605.

38. Musomba R, Mubiru F, Nakalema S, Mackline H, Kalule I, Kiragga AN, et al. Describing Point of Entry into Care and Being Lost to Program in a Cohort of HIV Positive Pregnant Women in a Large Urban Centre in Uganda. AIDS Res Treat. 2017;2017:3527563.

39. Mwapasa V, Pro G, Chinkhumba J, Mukaka M, Kobayashi E, Stuart A, et al. Mother-infant pair clinic and SMS messaging as innovative strategies for improving access to and retention in eMTCT care and Option B+ in Malawi: a cluster randomized control trial (the PRIME study). J Acquir Immune Defic Syndr. 2014 Nov 1;67 Suppl 2:S120-4.

40. Myer L, Phillips TK, Zerbe A, Brittain K, Lesosky M, Hsiao N-Y, et al. Integration of postpartum healthcare services for HIV-infected women and their infants in South Africa: A randomised controlled trial. PLoS Med. 2018 Mar;15(3):e1002547.

41. Nance N, Pendo P, Masanja J, Ngilangwa DP, Webb K, Noronha R, et al. Short-term effectiveness of a community health worker intervention for HIV-infected pregnant women in Tanzania to improve treatment adherence and retention in care: A cluster-randomized trial. PLoS One. 2017;12:e0181919.

42. Odeny TA, Hughes JP, Bukusi EA, Akama E, Geng E, Holmes K, et al. Text messaging for retention in PMTCT: A stepped-wedge cluster-randomized trial. Topics in Antiviral Medicine. 2018;26((Geng E.) University of California San Francisco, San Francisco, CA, United States):365s.

43. Oliver C, Rebeiro PF, Hopkins MJ, Byram B, Carpenter L, Clouse K, et al. Substance Use, Demographic and Socioeconomic Factors Are Independently Associated With Postpartum HIV Care Engagement in the Southern United States, 1999-2016. Open forum infectious diseases. 2019;6(2):ofz023.

44. Olwedo MA, Lukoda N, Crandall B. Retention of mother-baby pairs in care and treatment through mother-baby care point initiative in Eastern Uganda. Journal of the International AIDS Society. 2016;19((Olwedo M.A.; Lukoda N.; Crandall B.) STAR-E, Management Sciences for Health, Mbale, Uganda):232.

45. Oyeledun B, Phillips A, Oronsaye F, Alo OD, Shaffer N, Osibo B, et al. The Effect of a Continuous Quality Improvement Intervention on Retention-In-Care at 6 Months Postpartum in a PMTCT Program in Northern Nigeria: Results of a Cluster Randomized Controlled Study. Journal of acquired immune deficiency syndromes (1999). 2017;75 Suppl 2:S156–64.

46. Phiri S, Tweya H, Van Lettow M, Rosenberg NE, Trapence C, Kapito-Tembo A, et al. Impact of facility- and community-based peer support models on maternal uptake and retention in Malawi’s option B+ HIV prevention of mother-to-child transmission program: A 3-arm cluster randomized controlled trial (PURE Malawi). Journal of Acquired Immune Deficiency Syndromes. 2017;75((Chimbwandira F.) Department of HIV and AIDS, Ministry of Health, Lilongwe, Malawi):S140–8.

47. Puttkammer N, Domerçant JW, Adler M, Yuhas K, Myrtil M, Young P, et al. ART attrition and risk factors among Option B+ patients in Haiti: A retrospective cohort study. PloS one. 2017;12(3):e0173123.

48. Sarna A, Saraswati LR, Okal J, Matheka J, Owuor D, Singh RJ, et al. Cell Phone Counseling Improves Retention of Mothers With HIV Infection in Care and Infant HIV Testing in Kisumu, Kenya: A Randomized Controlled Study. Global health, science and practice. 2019;7(2):171–88.

49. Schwartz SR, Clouse K, Yende N, Van Rie A, Bassett J, Ratshefola M, et al. Acceptability and Feasibility of a Mobile Phone-Based Case Management Intervention to Retain Mothers and Infants from an Option B+ Program in Postpartum HIV Care. Matern Child Health J. 2015 Sep;19:2029–37.

50. Siddiqui R, Bell T, Sangi-Haghpeykar H, Minard C, Levison J. Predictive factors for loss to postpartum follow-up among low income HIV-infected women in Texas. AIDS patient care and STDs. 2014;28(5):248–53.

51. Swain CA, Smith LC, Nash D, Pulver WP, Lazariu V, Anderson BJ, et al. Postpartum Loss to HIV Care and HIV Viral Suppression among Previously Diagnosed HIV-Infected Women with a Live Birth in New York State. PloS one. 2016;11(8):e0160775.

52. Tariq S, Elford J, Chau C, French C, Cortina-Borja M, Brown A, et al. Loss to Follow-Up After Pregnancy Among Sub-Saharan Africa-Born Women Living With Human Immunodeficiency Virus in England, Wales and Northern Ireland: Results From a Large National Cohort. Sexually transmitted diseases. 2016;43(5):283–9.

53. Tweya H, Gugsa S, Hosseinipour M, Speight C, Ng’ambi W, Bokosi M, et al. Understanding factors, outcomes and reasons for loss to follow-up among women in Option B+ PMTCT programme in Lilongwe, Malawi. Tropical medicine & international health : TM & IH. 2014;19(11):1360–6.

54. Yotebieng M, Thirumurthy H, Moracco KE, Edmonds A, Tabala M, Kawende B, et al. Conditional Cash Transfers to Increase Retention in PMTCT Care, Antiretroviral Adherence, and Postpartum Virological Suppression: A Randomized Controlled Trial. J Acquir Immune Defic Syndr. 2016 Aug 1;72 Suppl 2:S124-9.

55. Clouse K, Vermund SH, Maskew M, Lurie MN, MacLeod W, Malete G, et al. Mobility and Clinic Switching Among Postpartum Women Considered Lost to HIV Care in South Africa. J Acquir Immune Defic Syndr. 2017 Apr 1;74:383–9.

56. Joint United Nations Programme on HIV/AIDS. UNAIDS Regions [Internet]. 2020 Dec [cited 2020 Dec 29]. Available from: https://www.unaids.org/en/regionscountries/regions

57. Doi SAR, Barendregt JJ, Khan S, Thalib L, Williams GM. Advances in the meta-analysis of heterogeneous clinical trials I: The inverse variance heterogeneity model. Contemp Clin Trials. 2015 Nov;45(Pt A):130–8.

58. Barendregt JJ, Doi SA, Lee YY, Norman RE, Vos T. Meta-analysis of prevalence. J Epidemiol Community Health. 2013 Nov 1;67(11):974–8.
