# Supplementary material for: Updates to the Spectrum/AIM model for the UNAIDS 2020 HIV estimates
Source: J Int AIDS Soc. 2021 Sep 21;24(Suppl 5):e25778. doi: 10.1002/jia2.25778 (PMC8454674; doi:10.1002/jia2.25778)
Supplement: Supplementary file 2 — Appendix S2. Updates to breastfeeding patterns in sub‐Saharan Africa for the 2020 round of HIV estimates [file JIA2-24-e25778-s002.docx]

Supplementary Appendix 2: Updates to breastfeeding patterns in sub-Saharan Africa for the 2020 round of HIV estimates

# Surveys used

We estimated breastfeeding patterns by region of sub-Saharan Africa using nationally representative household surveys that included HIV testing. Table S1 lists the surveys included in our analysis. R code for this analysis is available at <https://github.com/rlglaubius/InfantFeedingAnalysis>. This repository uses the rdhs package in R [1] to access survey microdata from the Demographic and Health Surveys (DHS) Program [2], but excludes five surveys we included in our analysis that are not part of the DHS Program (2007 and 2012 AIDS Indicator Surveys in Kenya and Population-based HIV Impact Assessment surveys in Malawi, Zambia, and Zimbabwe). Microdata from these five surveys are available from Kenya’s National Bureau of Statistics [3] or upon request from the PHIA project [4].

# Sensitivity analysis

Our primary analysis found that breastfeeding practices changed substantially over time in Southern Africa but were relatively stable in other regions of sub-Saharan Africa. There may be some risk of overfitting in Southern Africa since data were available from just six household surveys with HIV testing, whereas nine to 28 surveys were available in other regions (Table S1). Further, the only household survey data available for South Africa were from a DHS conducted in 2016, while other surveys in the region were done earlier (2006-2014).

We conducted a sensitivity analysis to investigate whether our primary analysis may have confounded potential differences in breastfeeding practices between South Africa and other countries in Southern Africa with differences in breastfeeding practices over time. We compared our primary analysis, which included all surveys listed in Table S1, to a secondary analysis that that excluded South Africa’s 2016 DHS.

Average breastfeeding patterns among HIV-positive mothers in Southern Africa do differ by inclusion or exclusion of South Africa’s 2016 DHS (Figure S1). Inclusion of South Africa’s DHS results in lower point estimates of the proportion breastfeeding among HIV-positive mothers, and among HIV-negative mothers in about the first year after delivery. Differences in point estimates of breastfeeding among HIV-positive mothers differ more in 2005, while these differences narrow in more recent years.

Our comparison suggests temporal effect estimates for Southern Africa are sensitive to inclusion or exclusion of South Africa’s 2016 DHS. This indicates the potential for conflation of spatial (national) and temporal effects. Future household surveys in the region may allow more reliable estimation of these parameters. While Population-based HIV Impact Assessment (PHIA) surveys done in Malawi, Zambia, and Zimbabwe elicited breastfeeding status via questions analogous to those used in Demographic and Health Surveys and AIDS Indicator Surveys, later PHIAs did not, including those conducted in Eswatini (2016-17), Lesotho (2016-18), and Namibia (2017). However, we may be able to incorporate more recent PHIAs in Eswatini and Lesotho once data from those surveys become available.

Table S1. Household surveys included in analyses of breastfeeding patterns.

| Country | Surveys |
| --- | --- |
| **Central Africa** |  |
| Angola | 2015-16 DHS |
| Cameroon | 2004 DHS, 2011 DHS, 2018 DHS |
| Chad | 2014-15 DHS |
| Democratic Republic of the Congo | 2007 DHS, 2013-14 DHS |
| Gabon | 2012 DHS |
| Sao Tome and Principe | 2008-09 DHS |
|  |  |
| **Eastern Africa** |  |
| Burundi | 2010 DHS, 2016-17 DHS |
| Ethiopia | 2005 DHS, 2011 DHS, 2016 DHS |
| Kenya | 2003 DHS, 2007 AIS, 2008-09 DHS, 2012 AIS |
| Malawi | 2004 DHS, 2010 DHS, 2015-16 DHS, 2015-16 PHIA |
| Mozambique | 2015 AIS |
| Rwanda | 2005 DHS, 2010 DHS, 2014-15 DHS |
| United Republic of Tanzania | 2007-08 AIS, 2011-12 AIS |
| Uganda | 2011 AIS |
| Zambia | 2007 DHS, 2013-14 DHS, 2016 PHIA, 2018 DHS |
| Zimbabwe | 2005-06 DHS, 2010-11 DHS, 2015 DHS, 2015-16 PHIA |
|  |  |
| **Southern Africa** |  |
| Eswatini | 2006-07 DHS |
| Lesotho | 2004 DHS, 2009 DHS, 2014 DHS |
| Namibia | 2013 DHS |
| South Africa | 2016 DHS |
|  |  |
| **Western Africa** |  |
| Burkina Faso | 2003 DHS, 2010 DHS |
| Côte d’Ivoire | 2011-12 DHS |
| The Gambia | 2013 DHS |
| Ghana | 2003 DHS, 2014 DHS |
| Guinea | 2005 DHS, 2012 DHS, 2018 DHS |
| Liberia | 2007 DHS, 2013 DHS |
| Mali | 2006 DHS, 2012-13 DHS |
| Niger | 2006 DHS, 2012 DHS |
| Senegal | 2005 DHS, 2010-11 DHS, 2017 DHS |
| Sierra Leone | 2008 DHS, 2013 DHS |
| Togo | 2013-14 DHS |

AIS, AIDS Indicator Survey; DHS, Demographic and Health Survey; PHIA, Population-Based HIV Impact Assessment


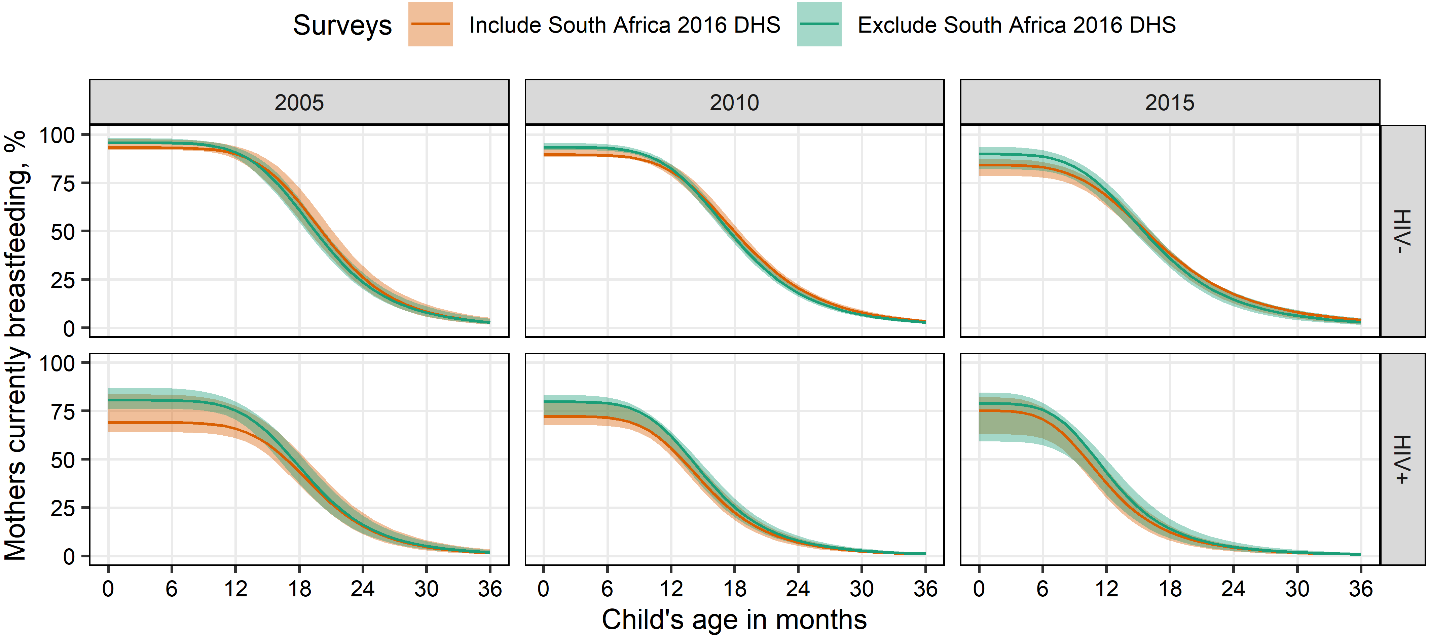


Figure S1. Modeled breastfeeding duration in Southern Africa by HIV status in 2005, 2010, and 2015. Trends are based on average country effects. Maximum likelihood point estimates (solid curves) and 95% central credible intervals (shaded areas) are shown for our main analysis that includes all surveys in Table S1 (orange) to secondary analysis that excludes South Africa’s 2016 DHS (green).

# Breastfeeding model fit to survey data

Figures on the next several pages show the breastfeeding model fitted to each Demographic Health Survey (DHS) and AIDS Indicator Survey (AIS) from our primary analysis. Model fits to data from Population-Based Impact Assessment (PHIA) surveys in Malawi (2015-16), Zambia (2016), and Zimbabwe (2015-16) are not shown because key data on child birth dates have been redacted from public datasets for privacy concerns. Figures are grouped by region and ordered alphabetically by country. For each survey, panels show the model fit to breastfeeding among HIV-negative mothers (left), HIV-positive mothers (center), and a comparison of modelled trends by HIV status (right). Solid curves show the posterior mode point estimate used by Spectrum/AIM. Solid regions show the 95% central credible interval about model estimates. Black points and error bars show survey point estimates and 95% confidence intervals, which we calculated using the “survey” package [5] in R using the function “svyciprop” with “logit” method. Confidence intervals around some survey estimates could not be calculated for some durations due to small sample sizes.

## Central Africa


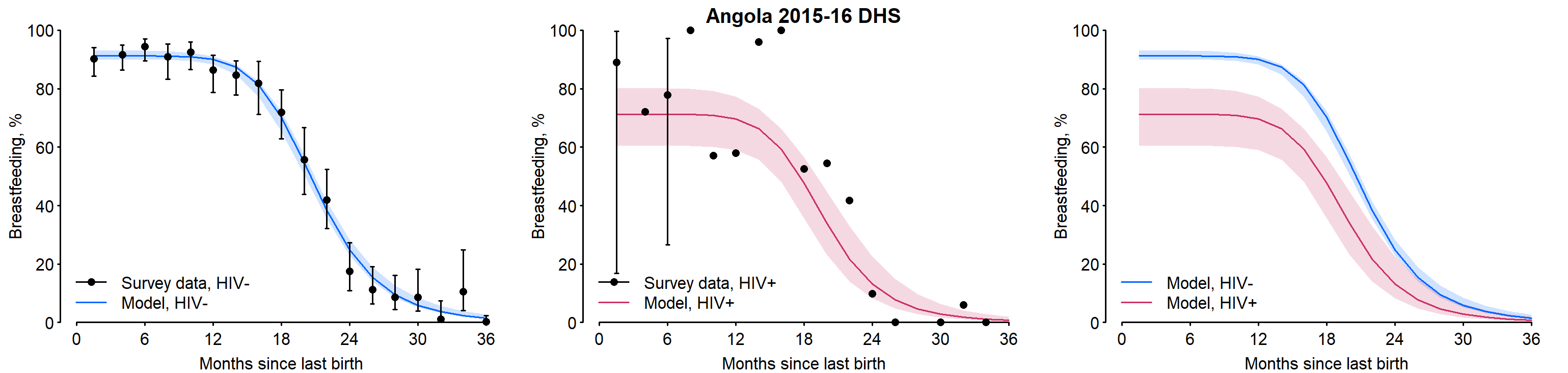


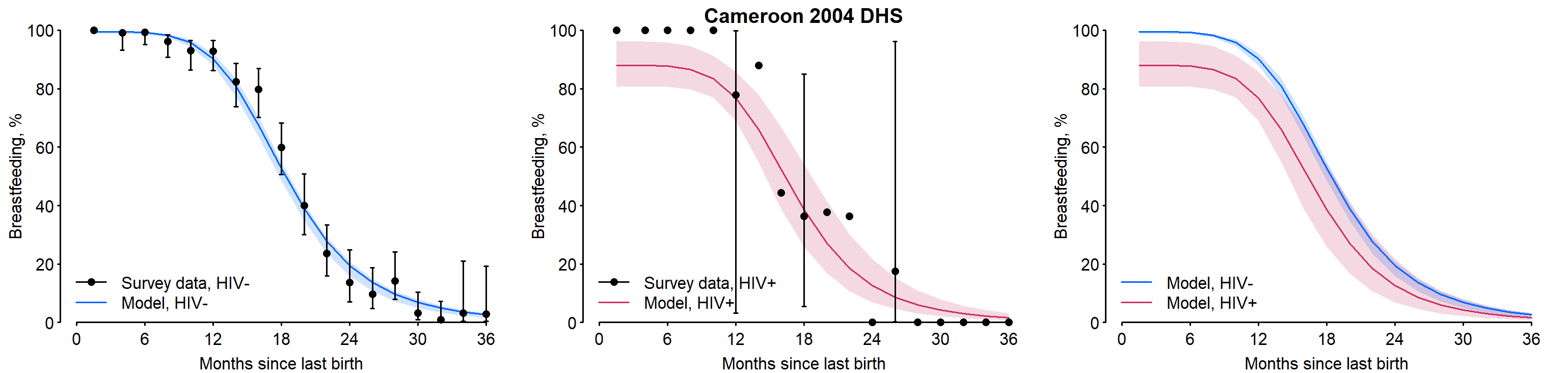


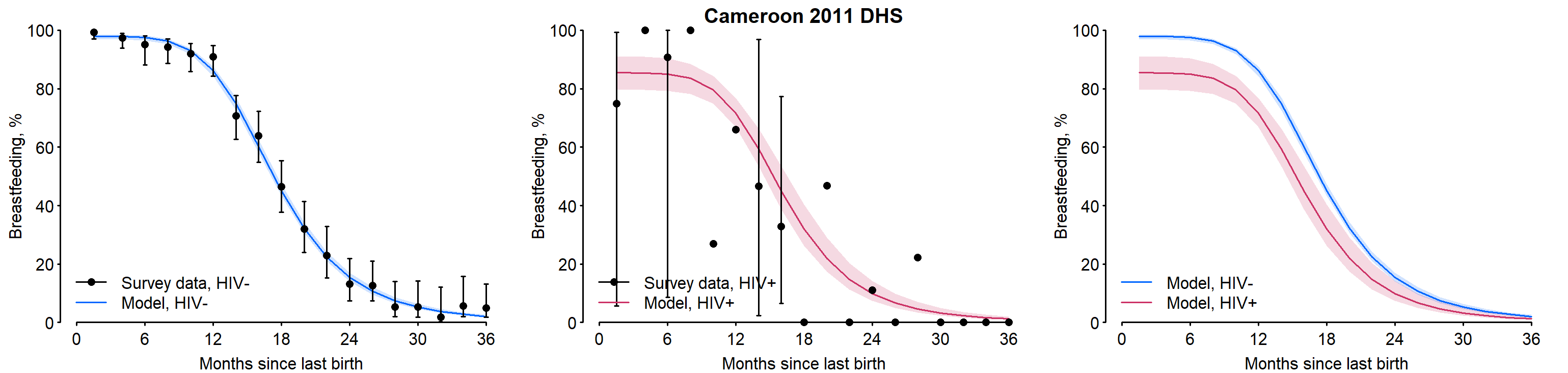


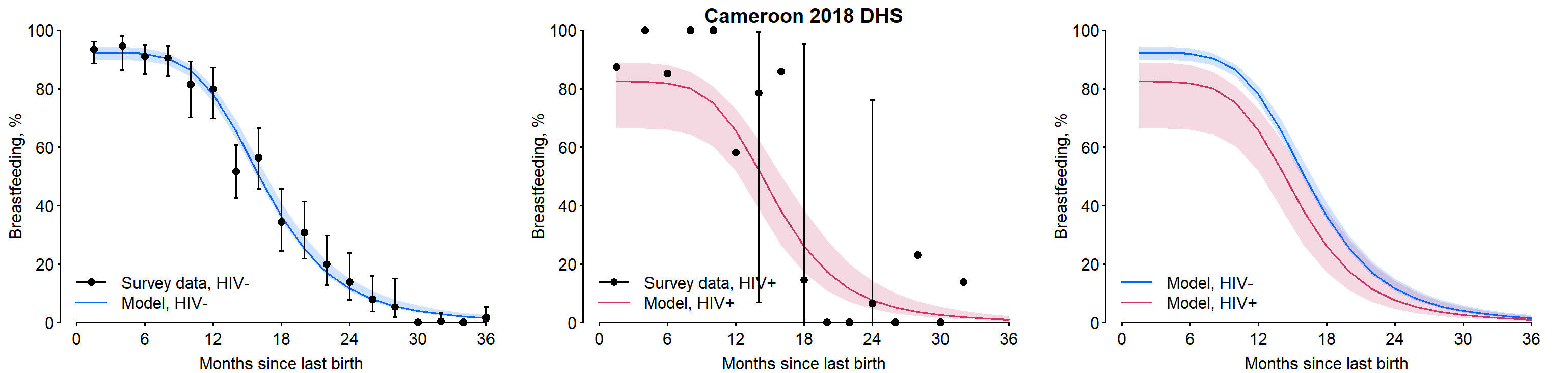

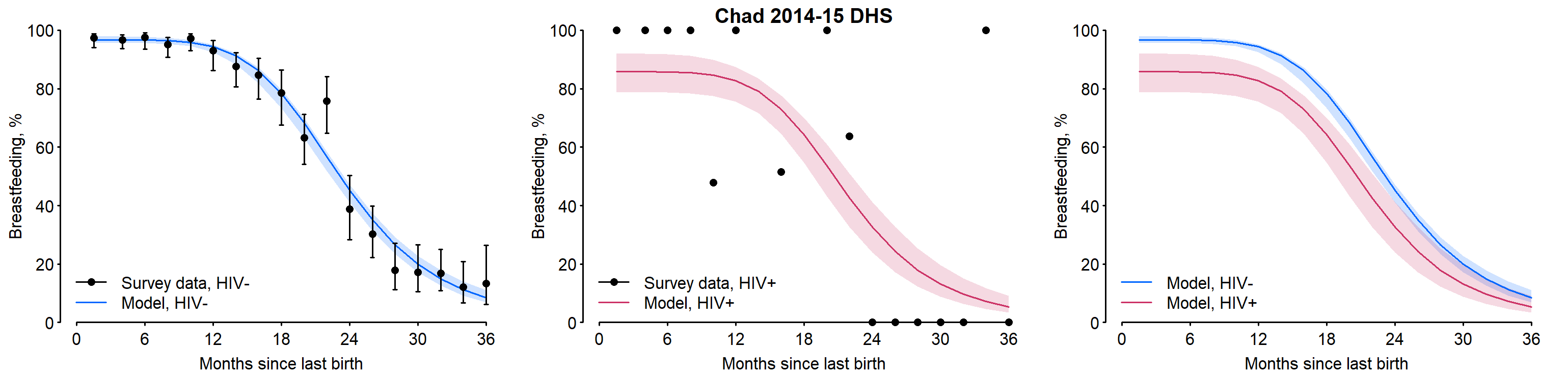


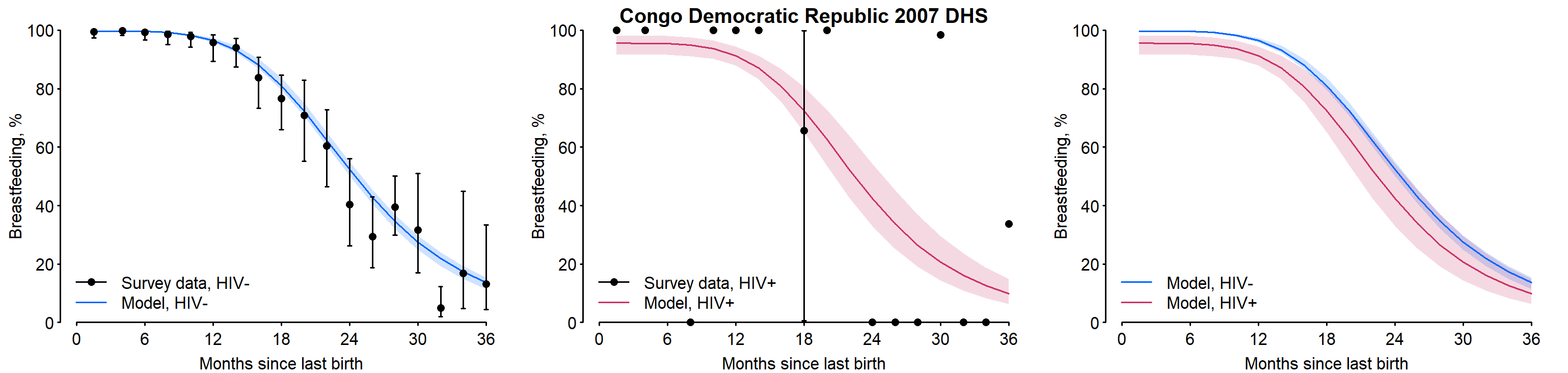

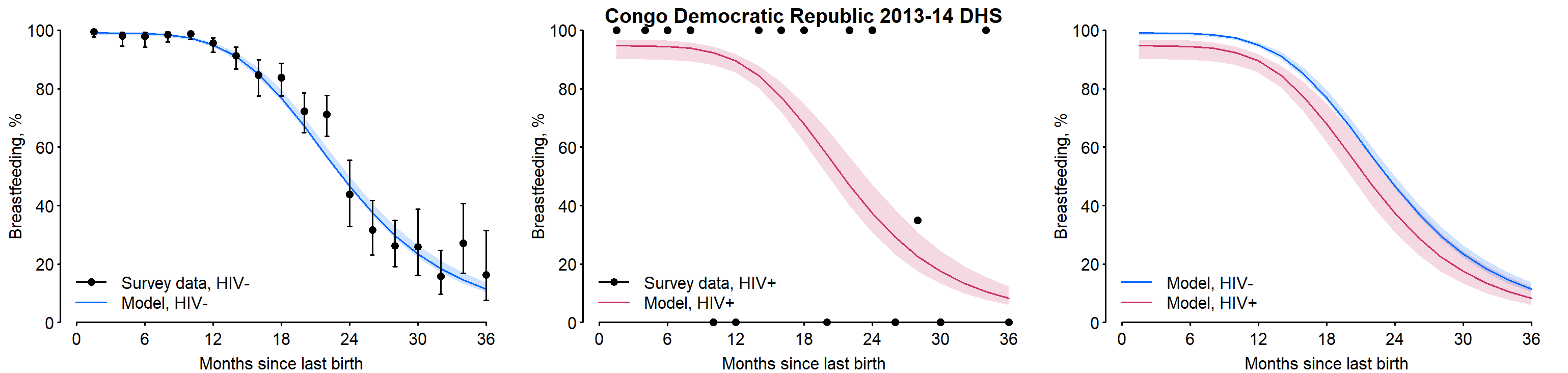

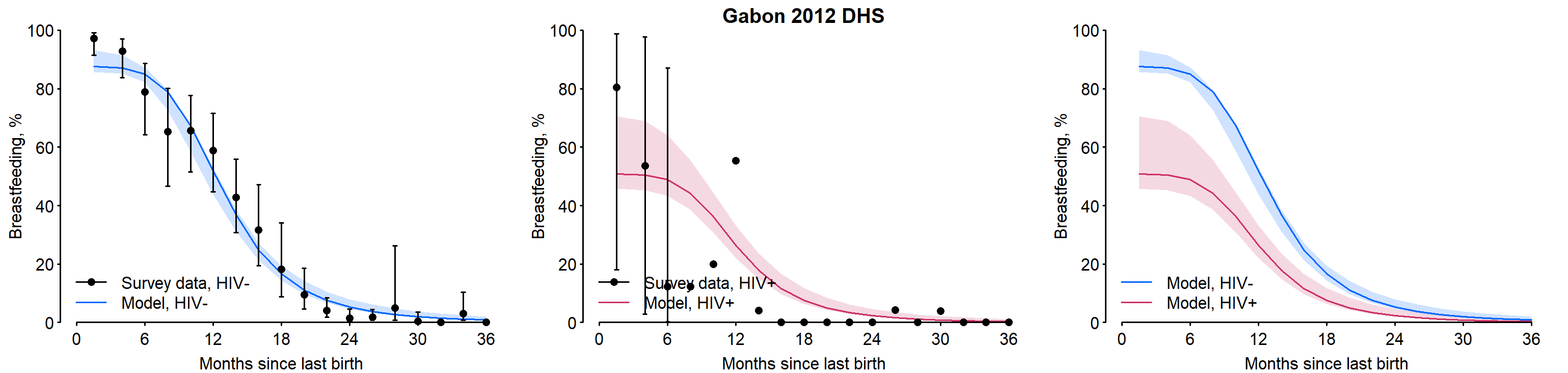

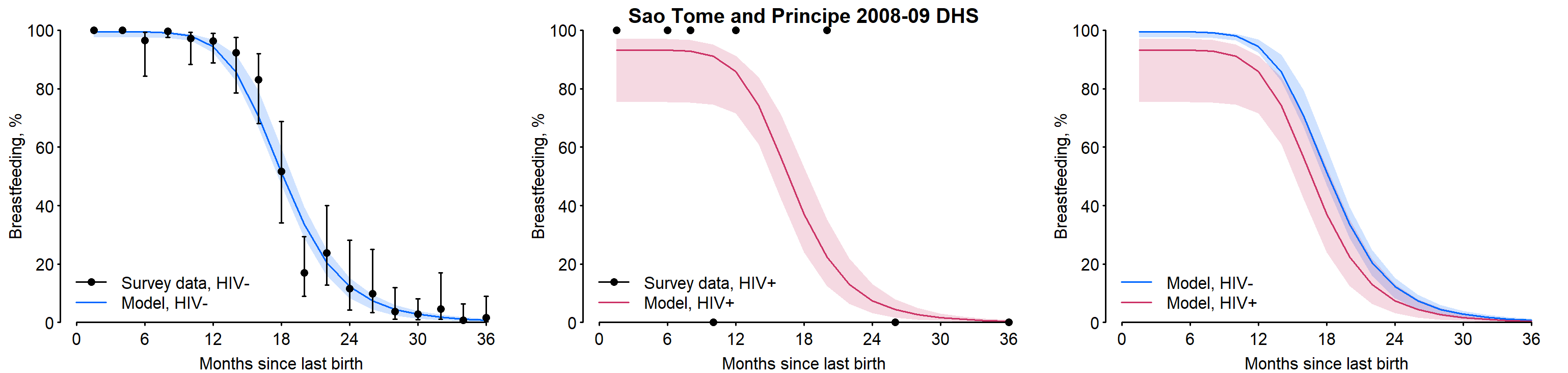


## Eastern Africa


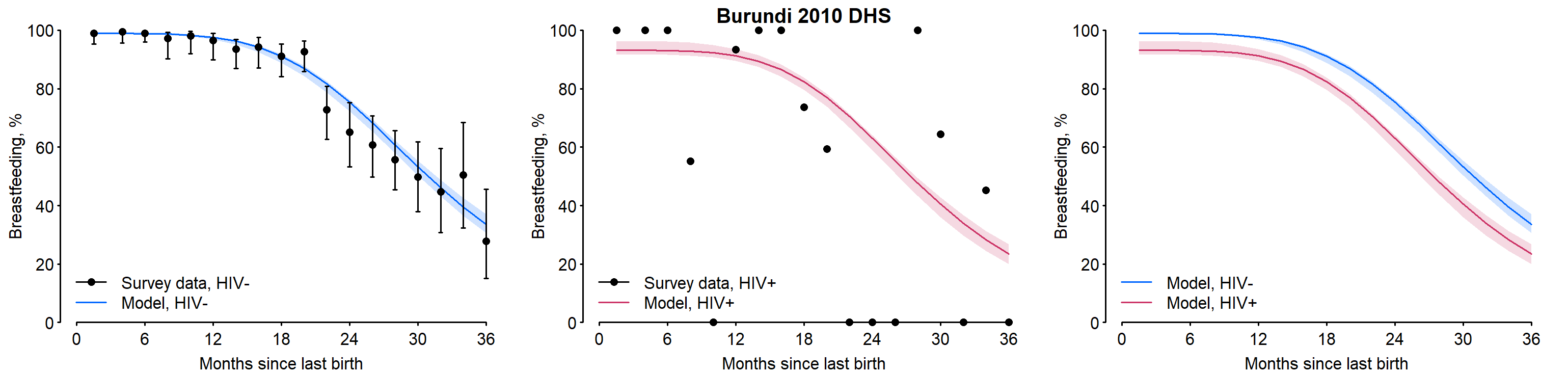


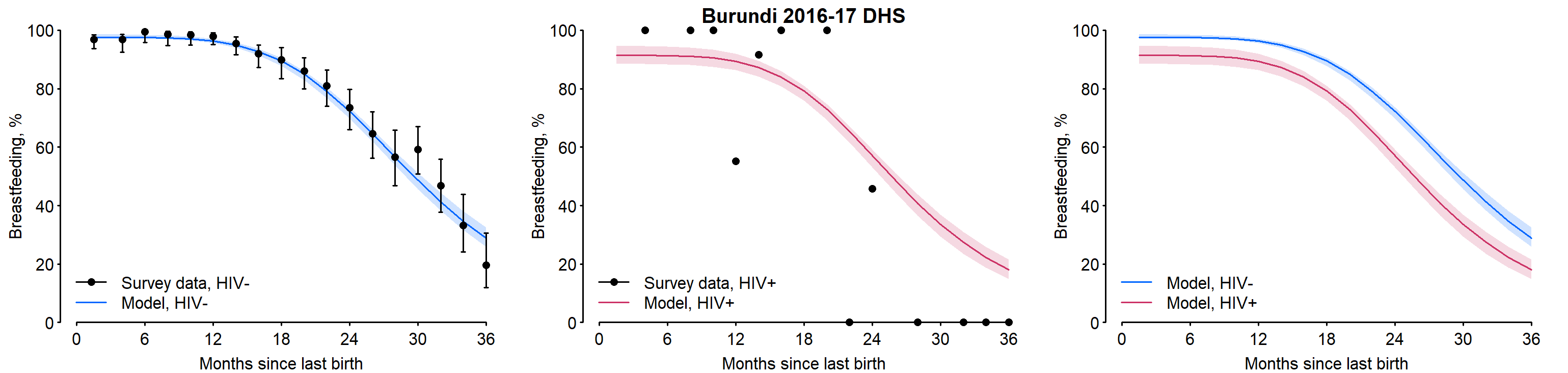

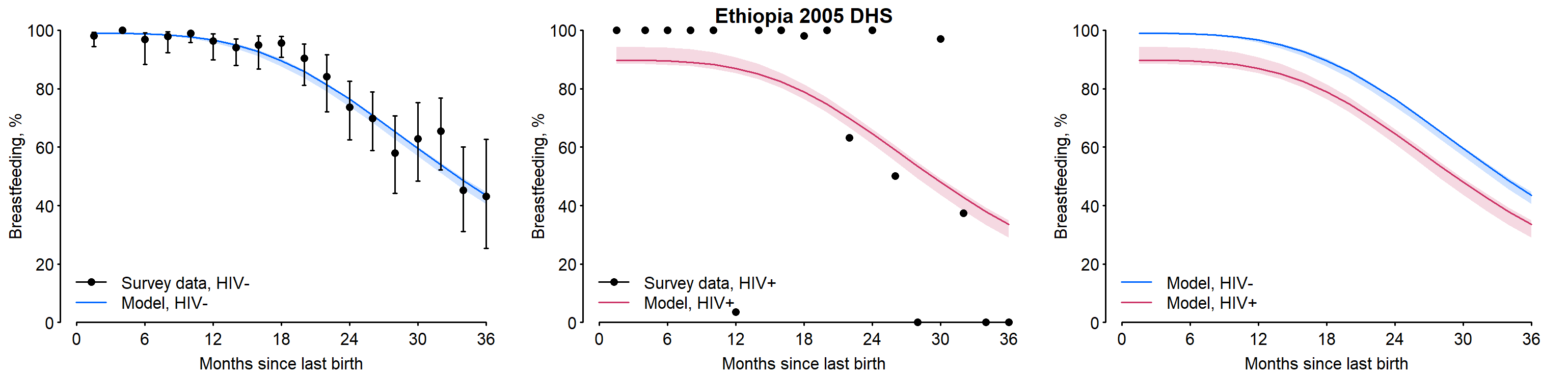

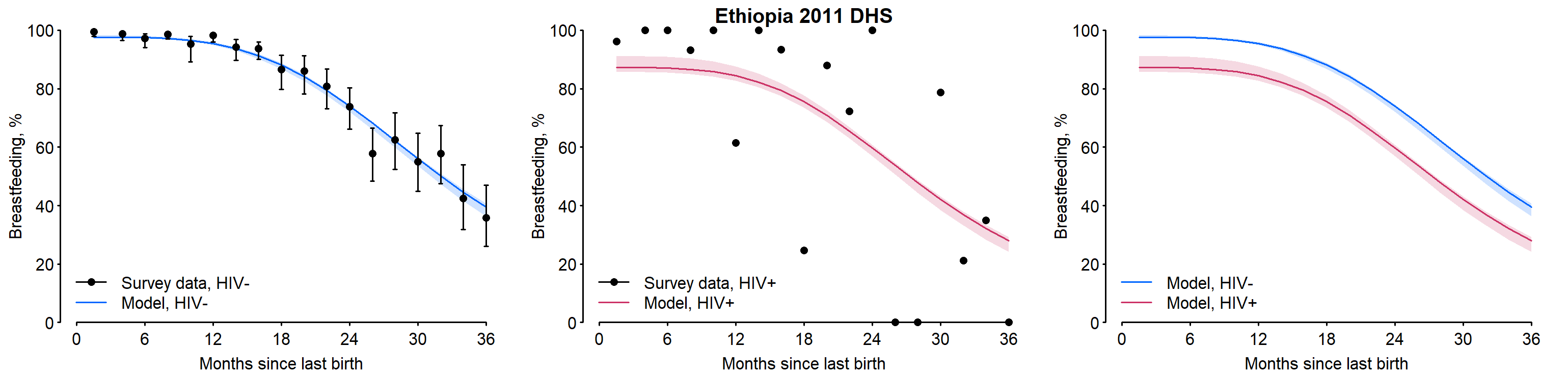

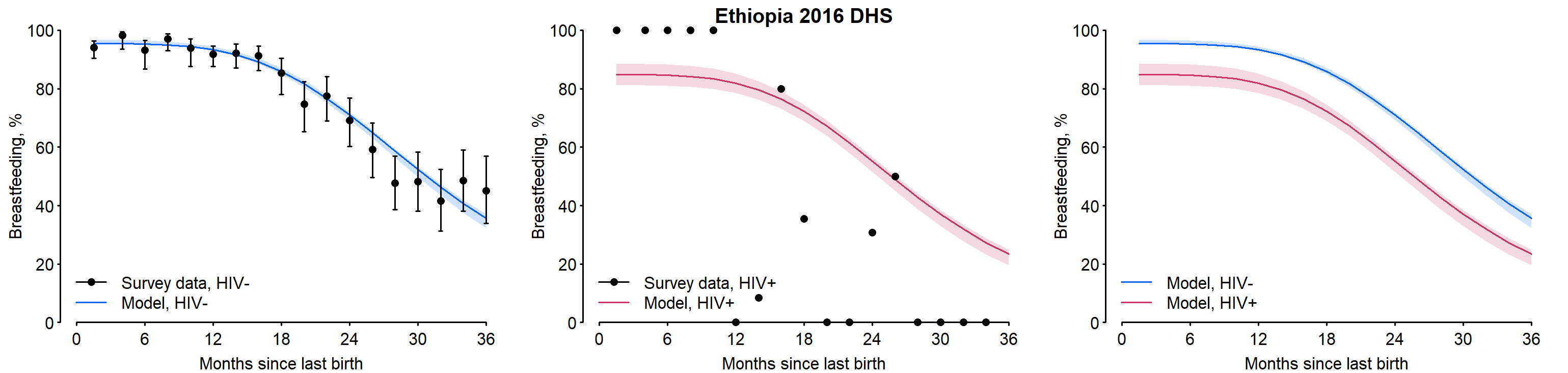

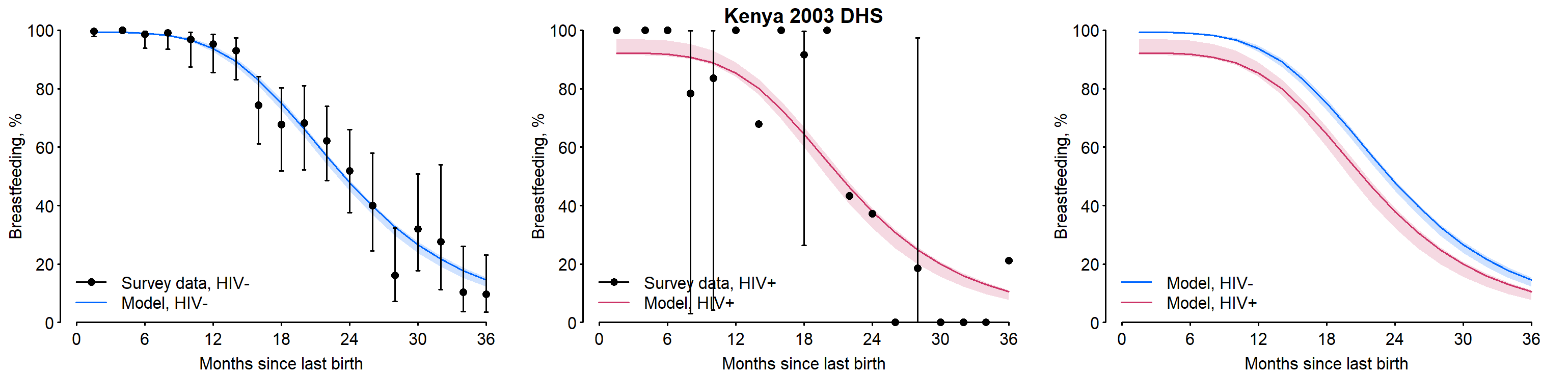

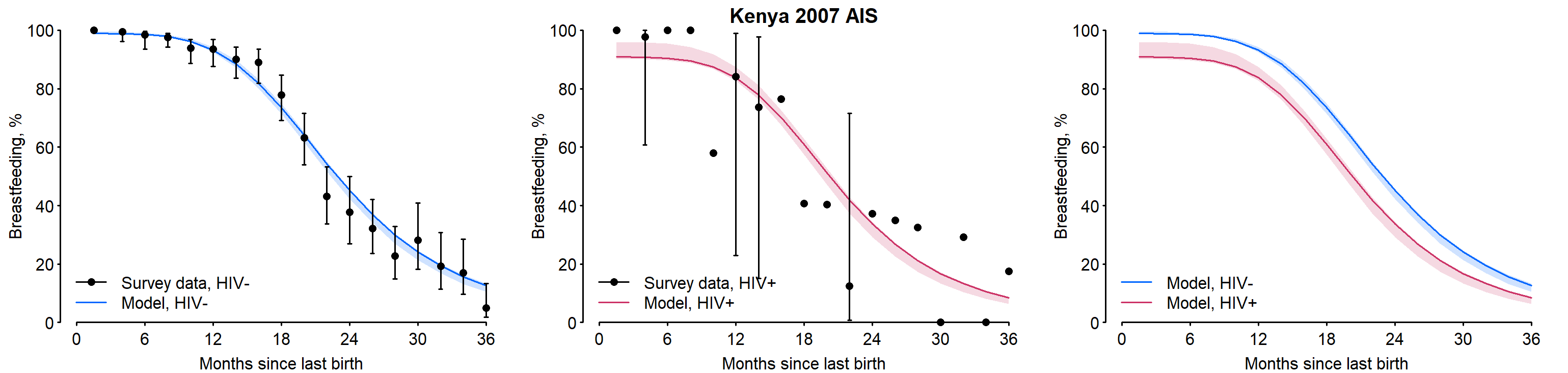

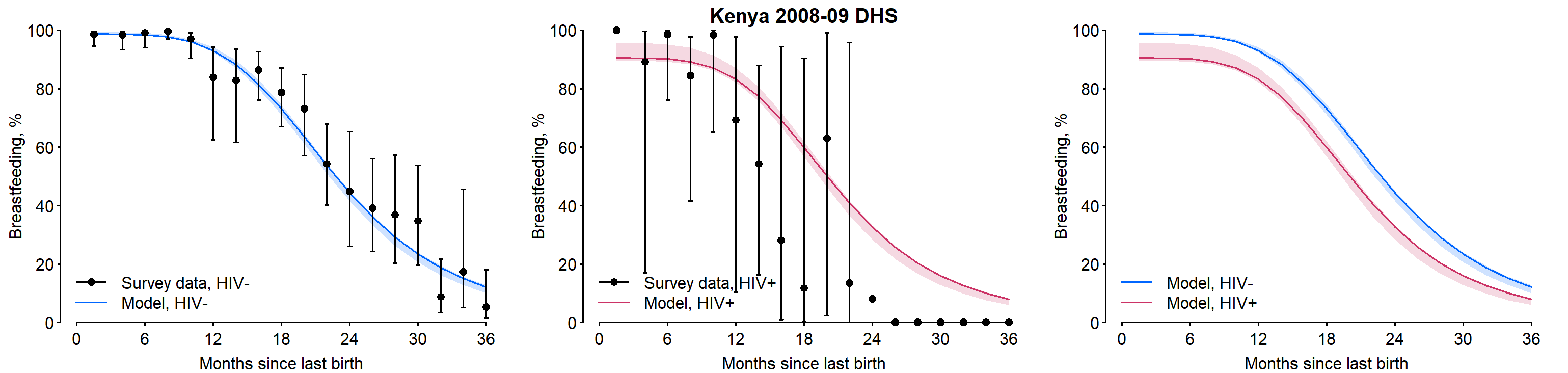

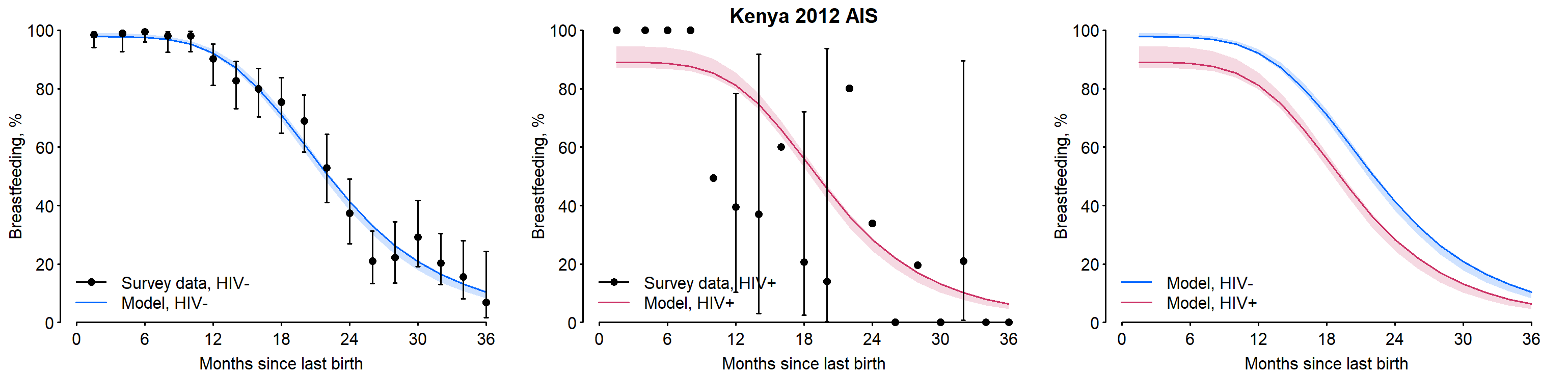

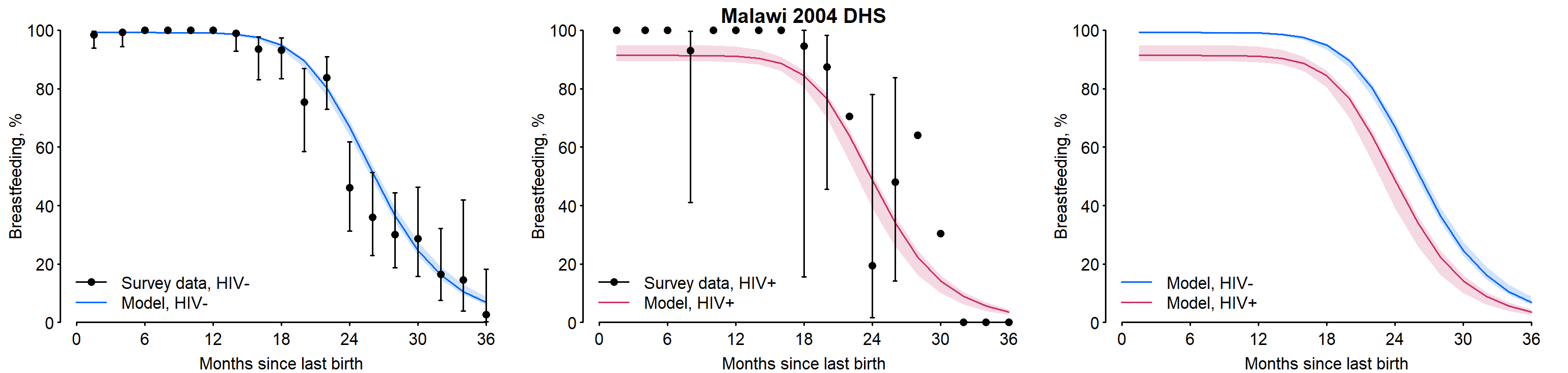

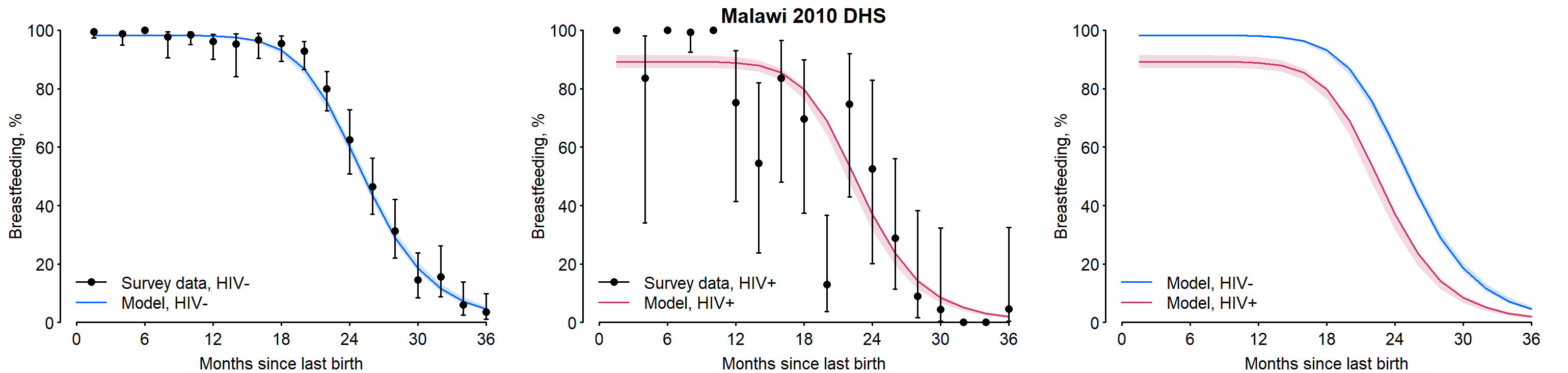

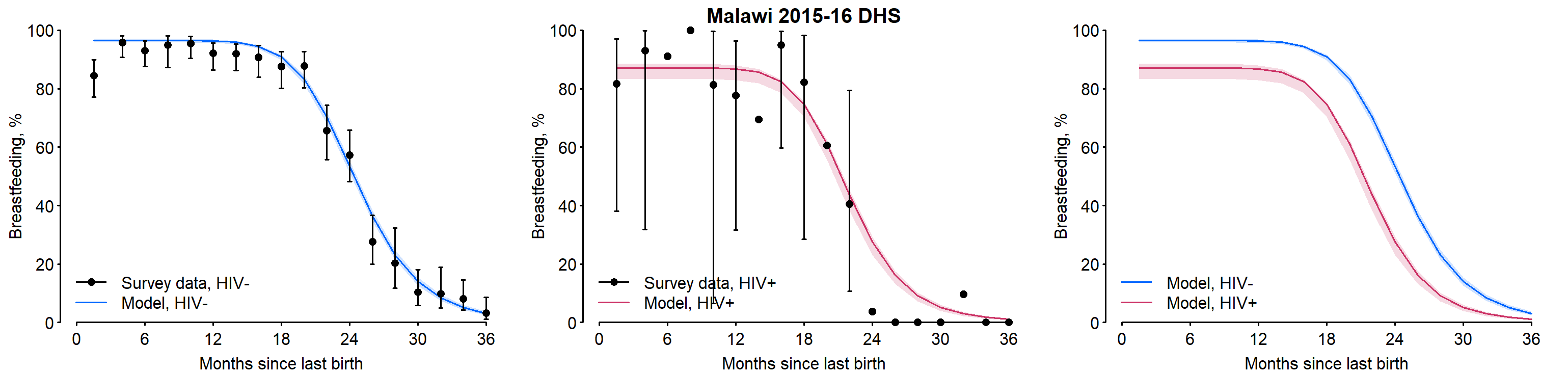

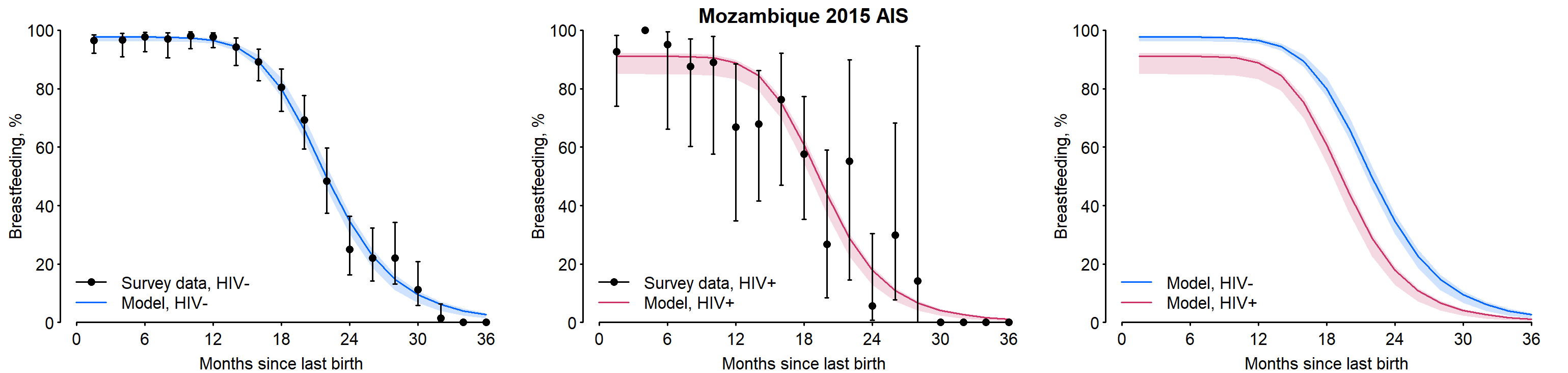

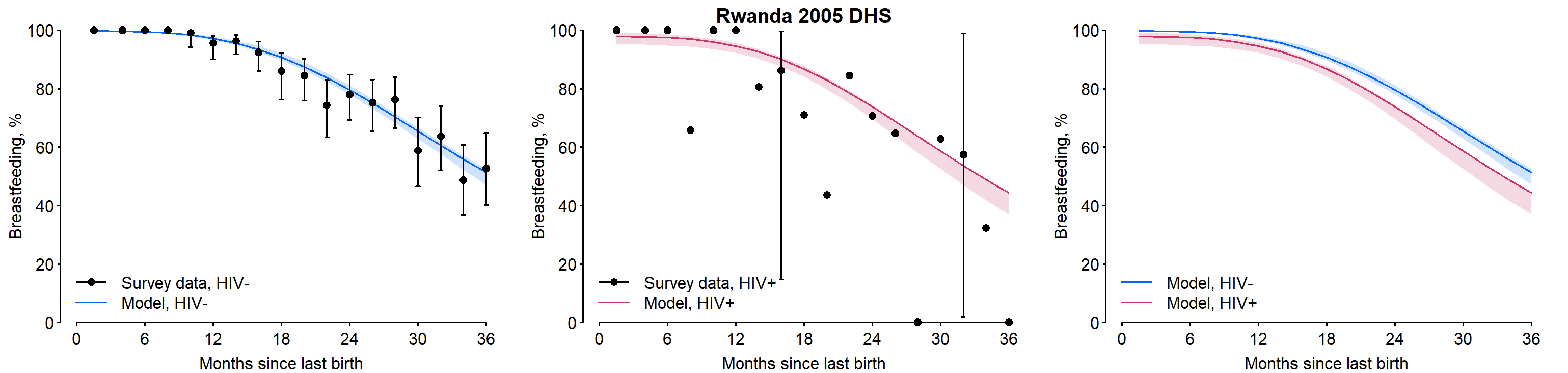

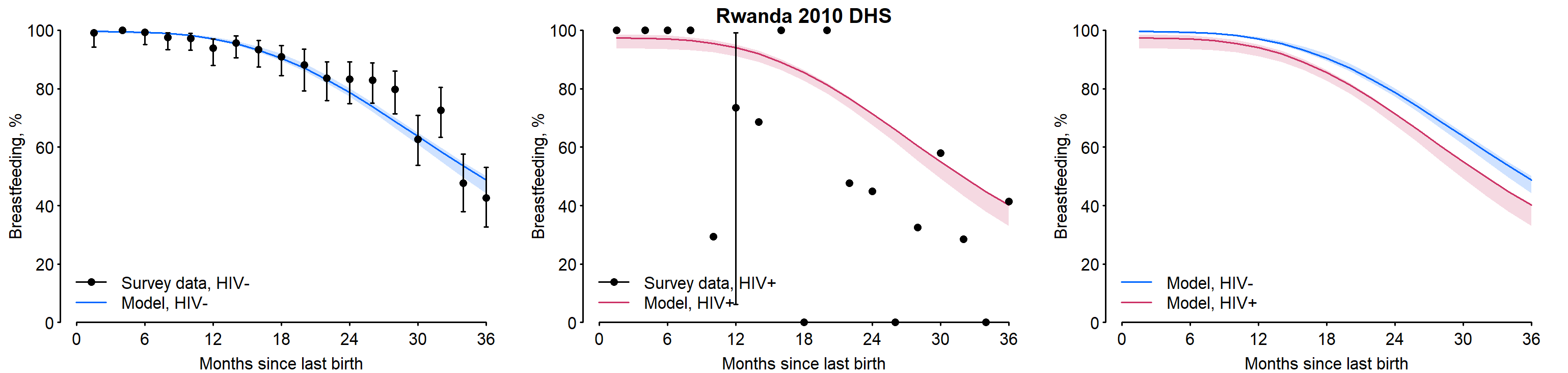

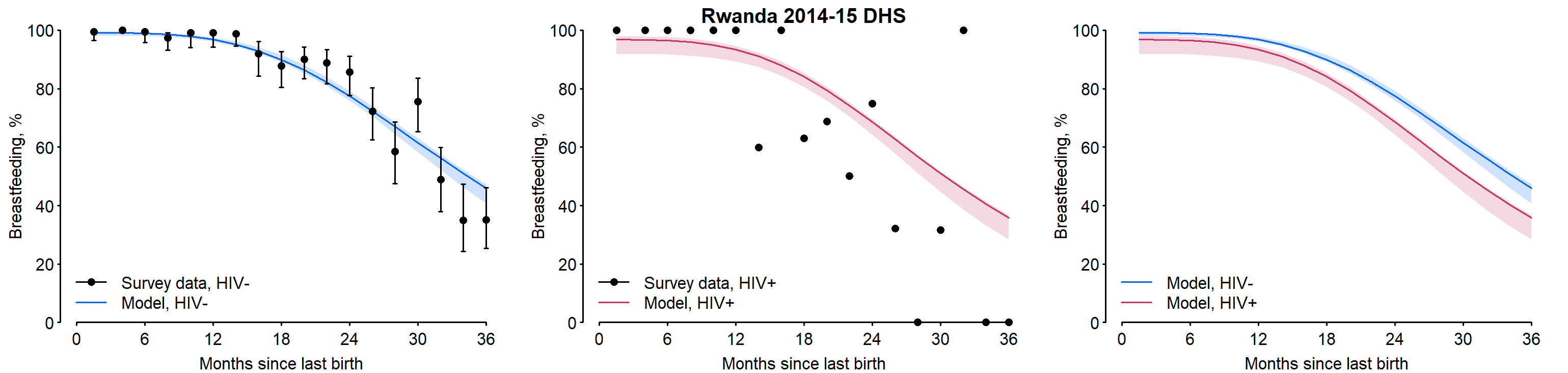

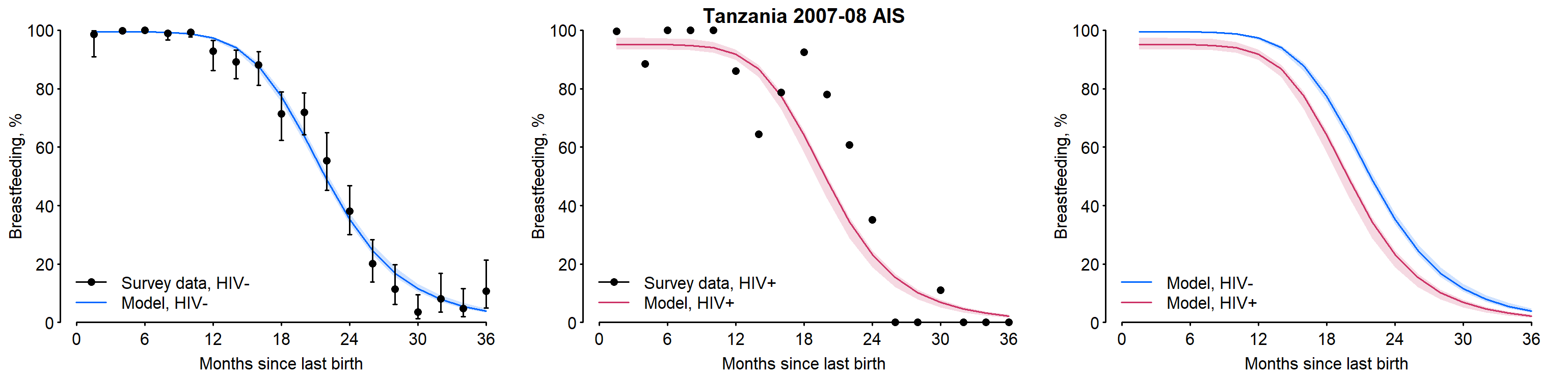

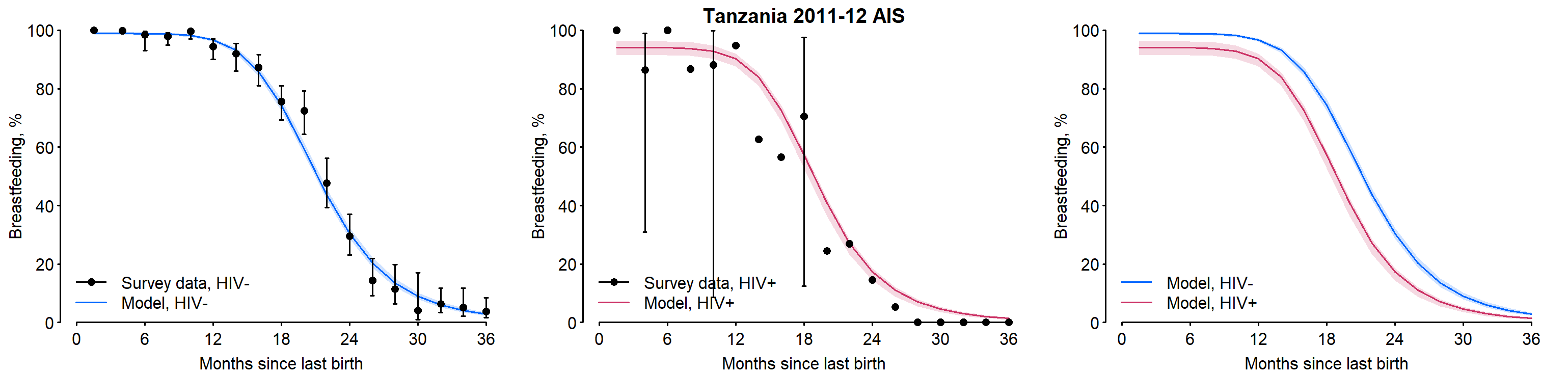

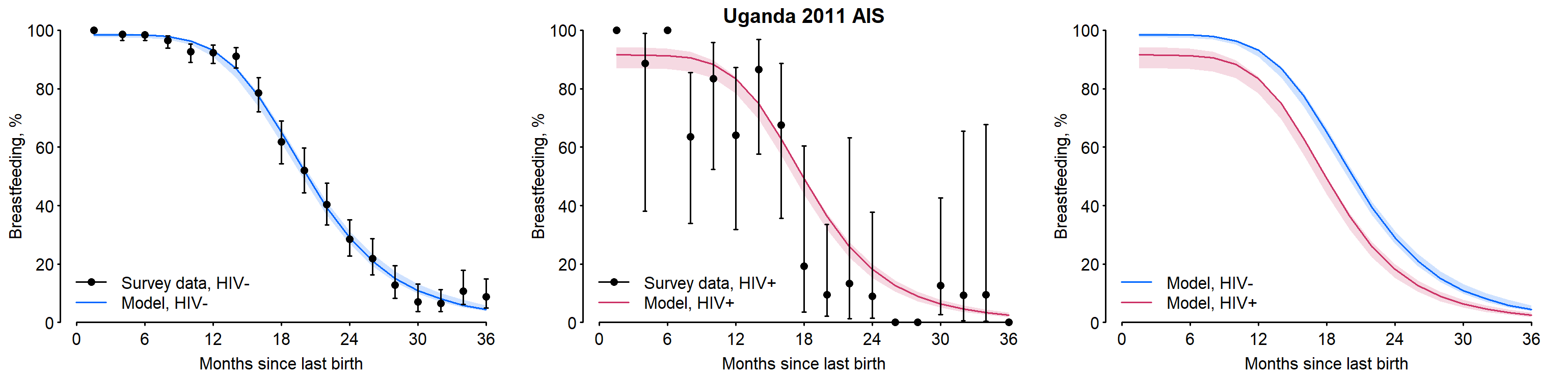

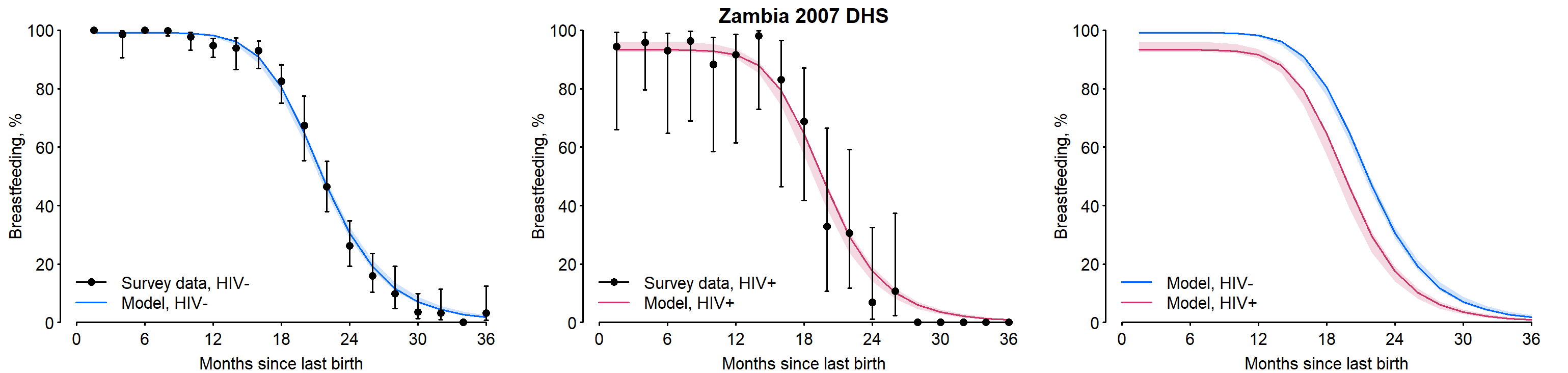

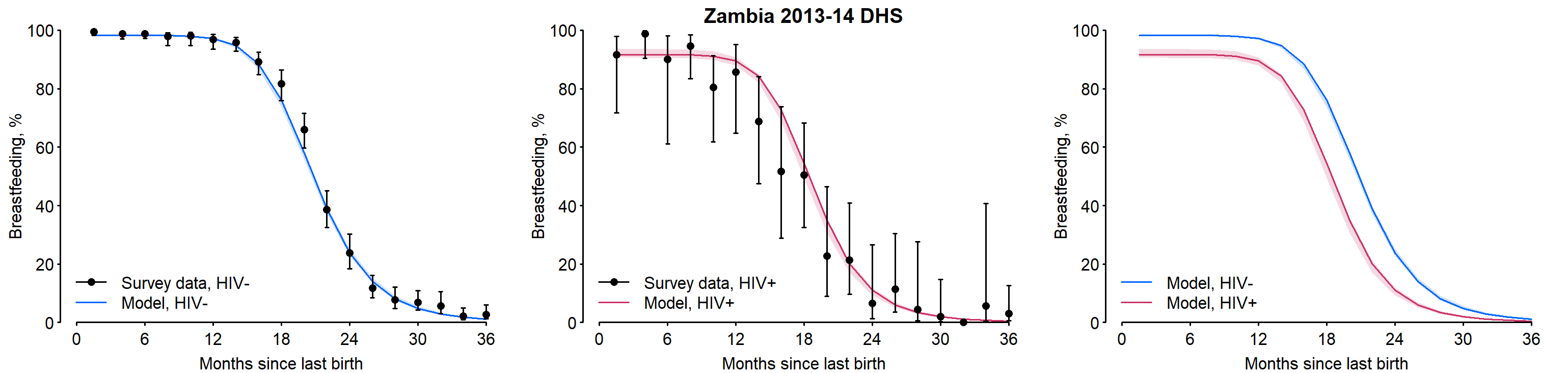

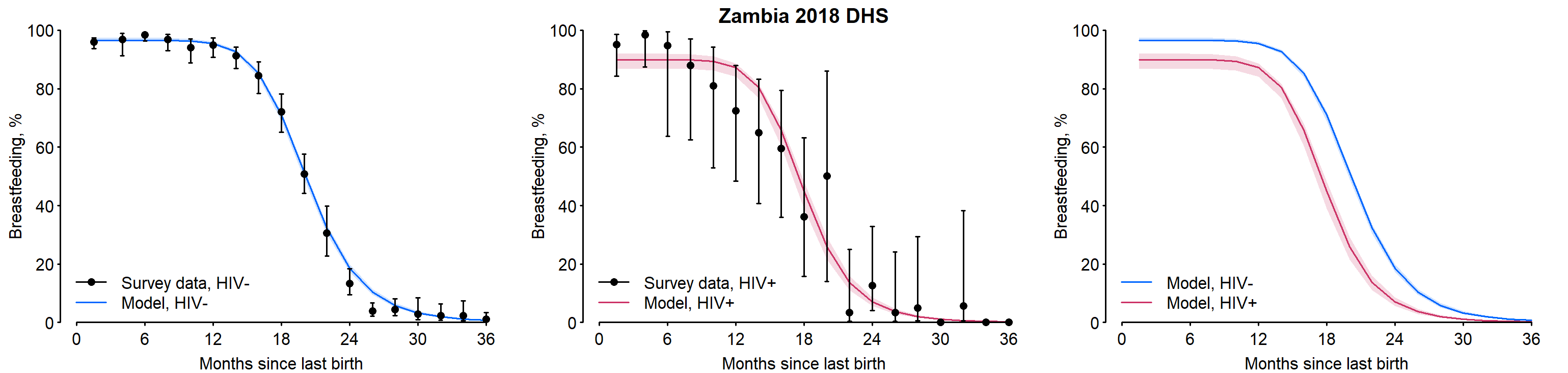

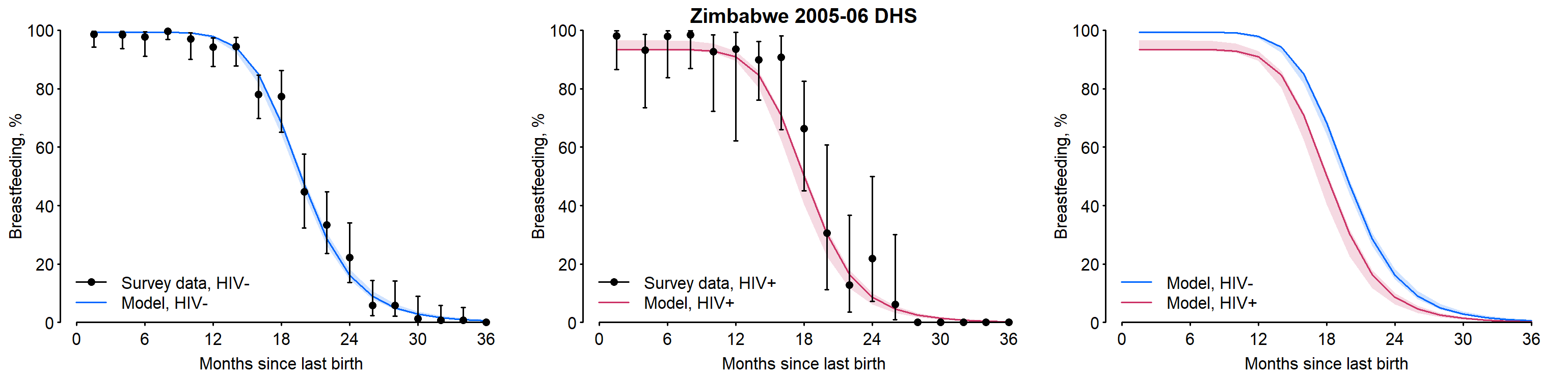

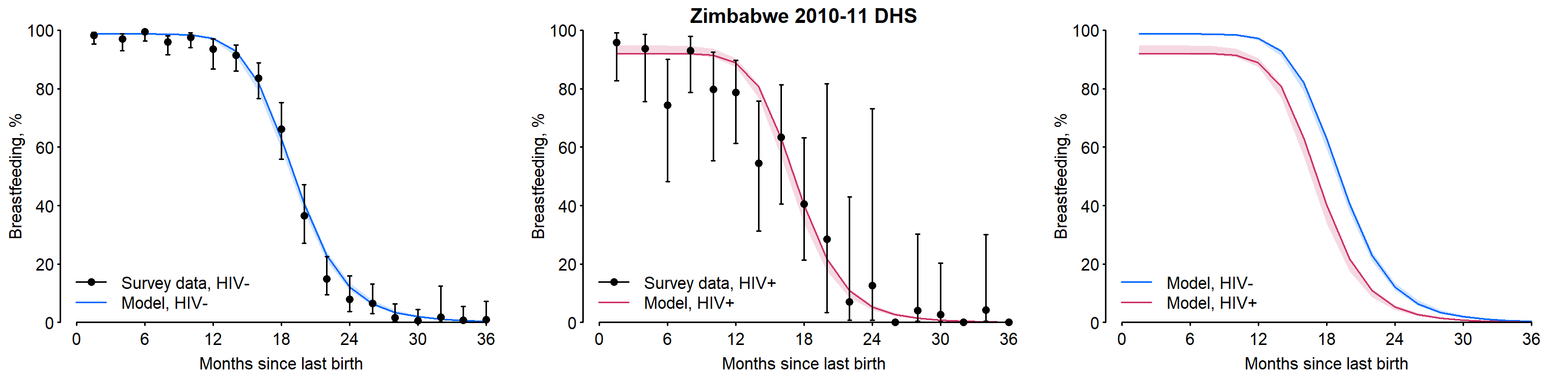

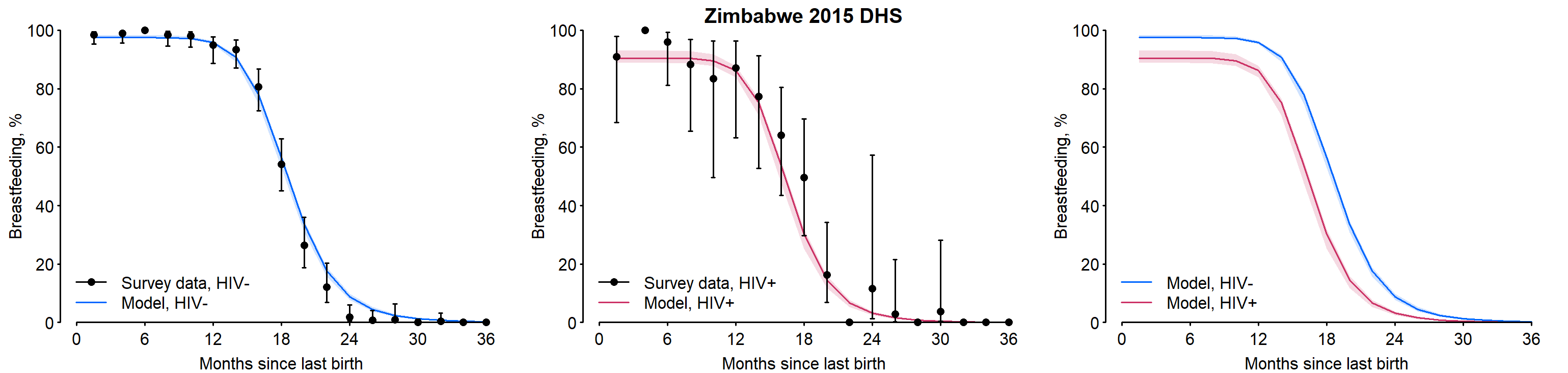


## Southern Africa
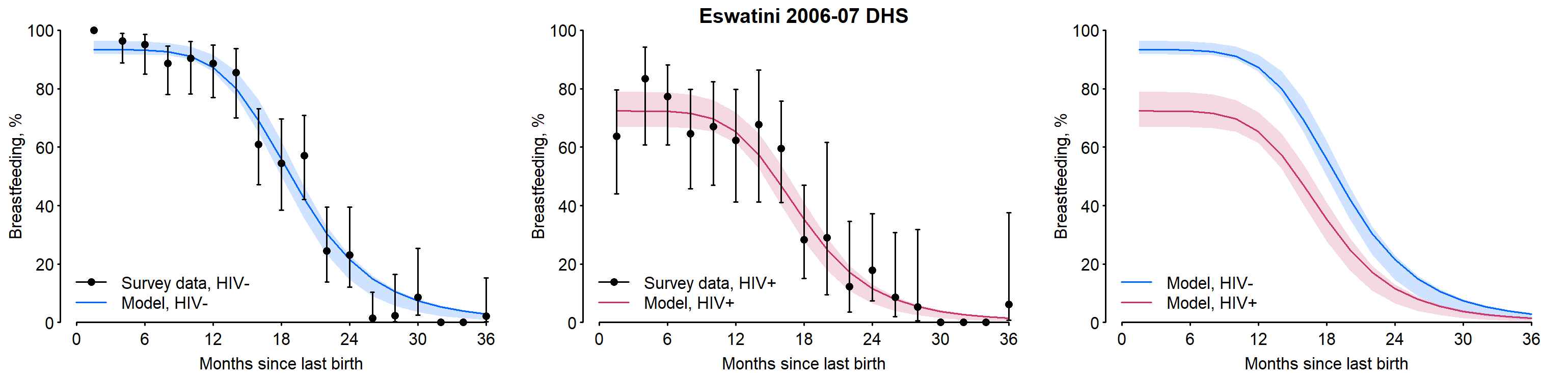

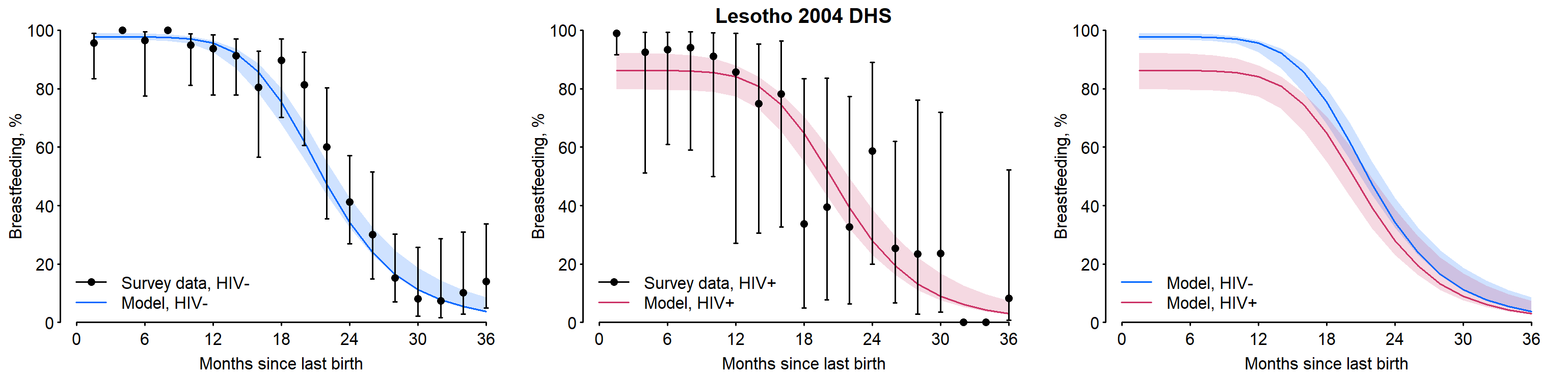

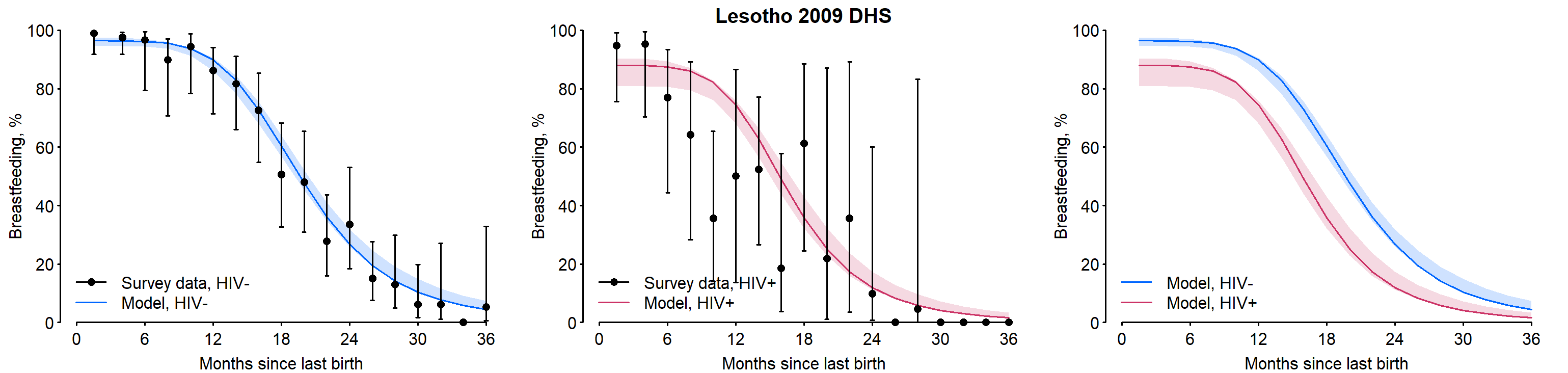

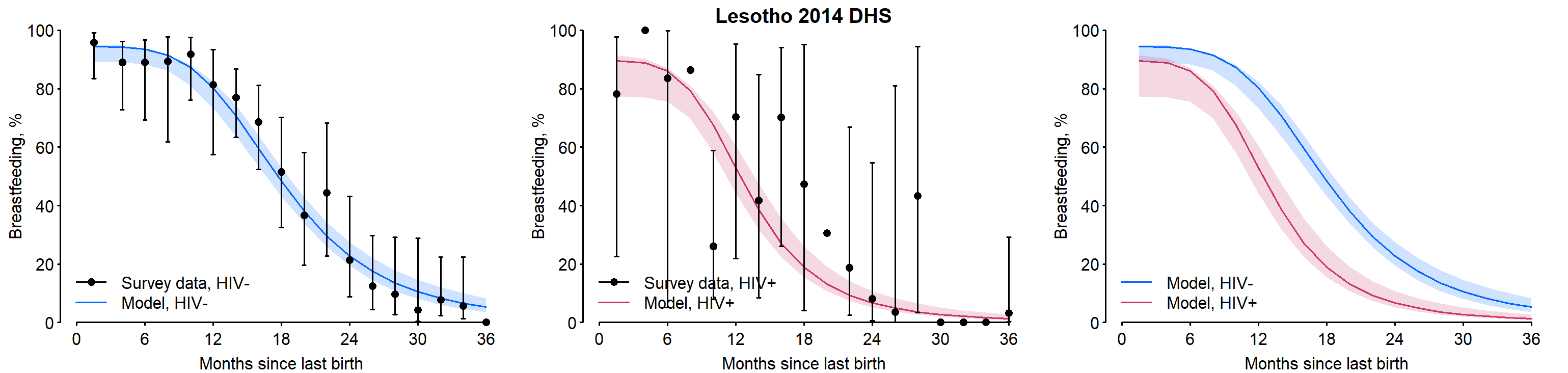

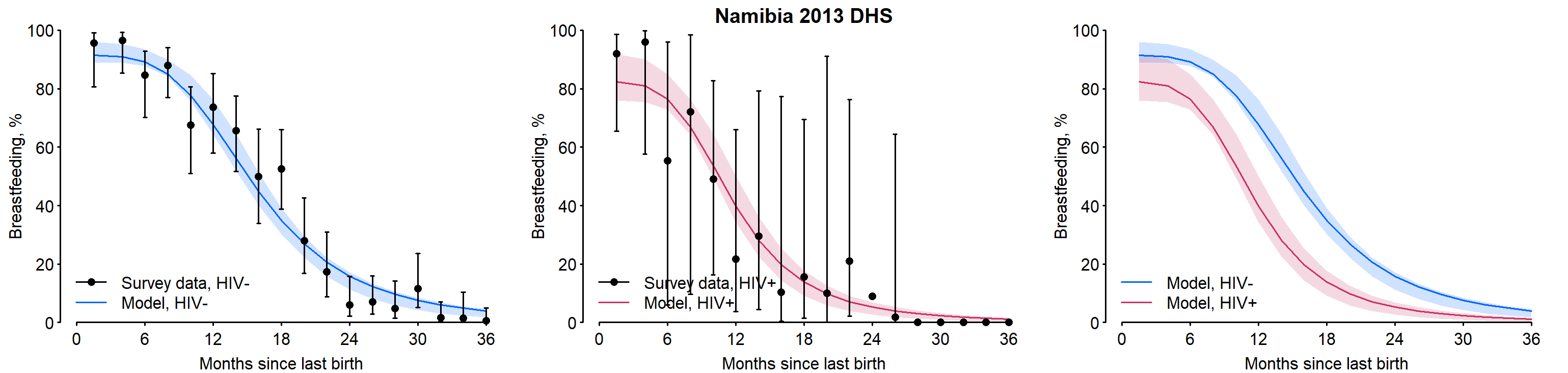

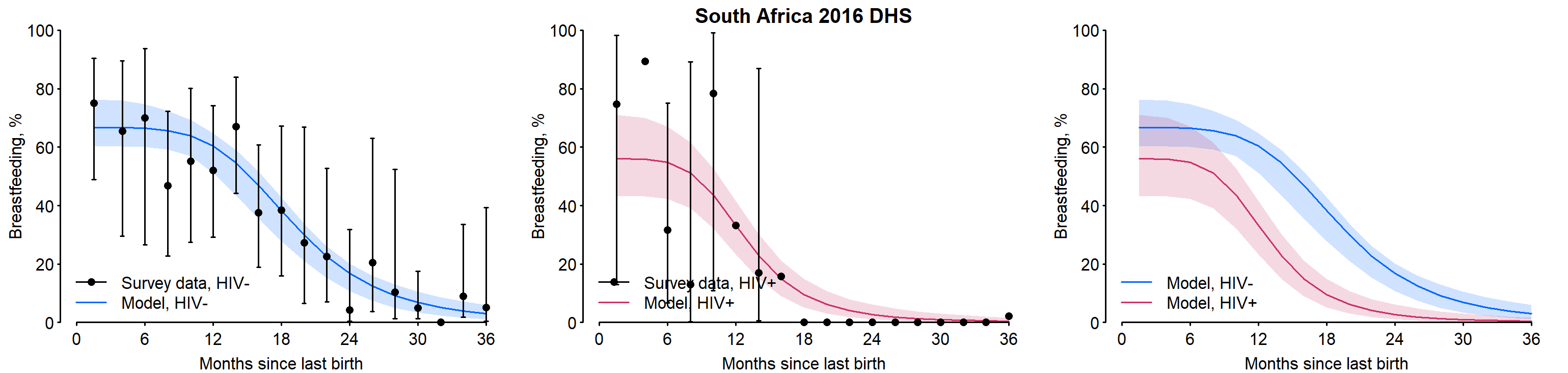


## Western Africa


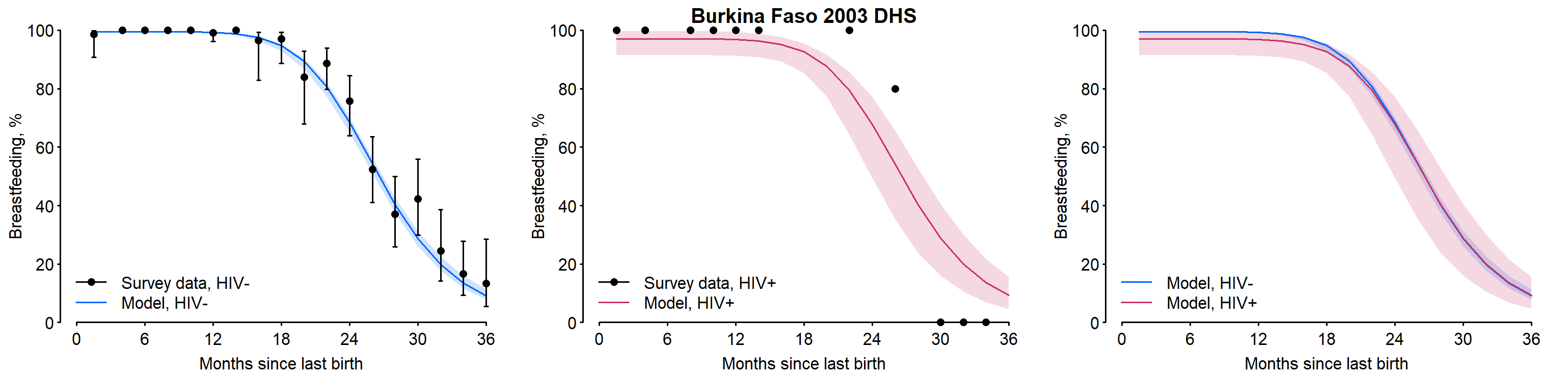


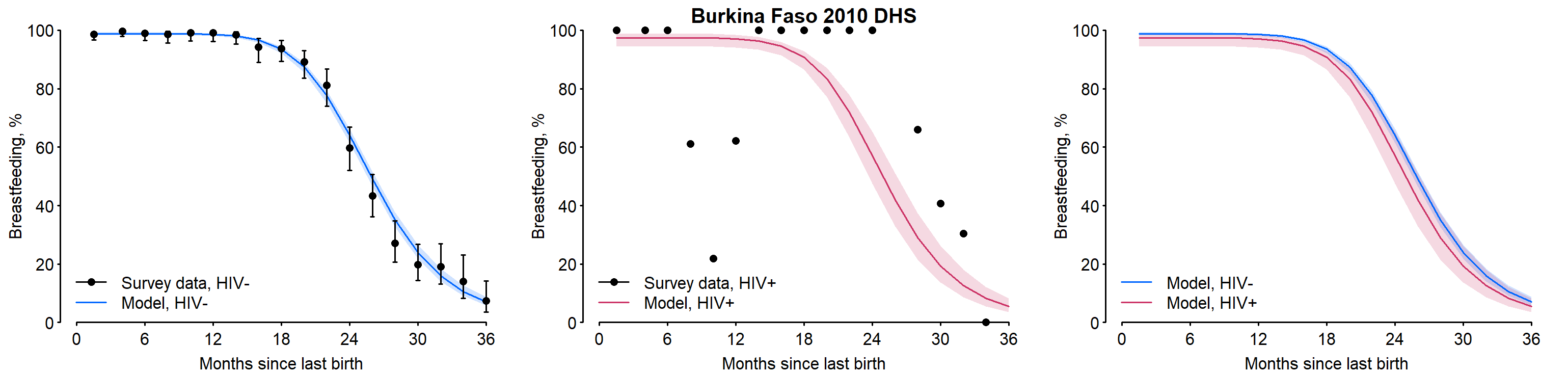


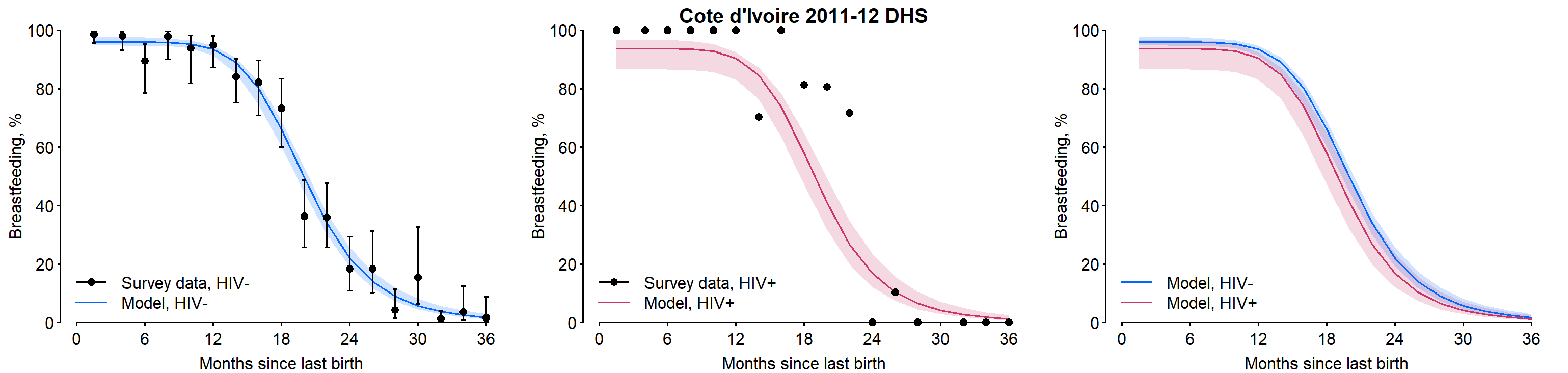

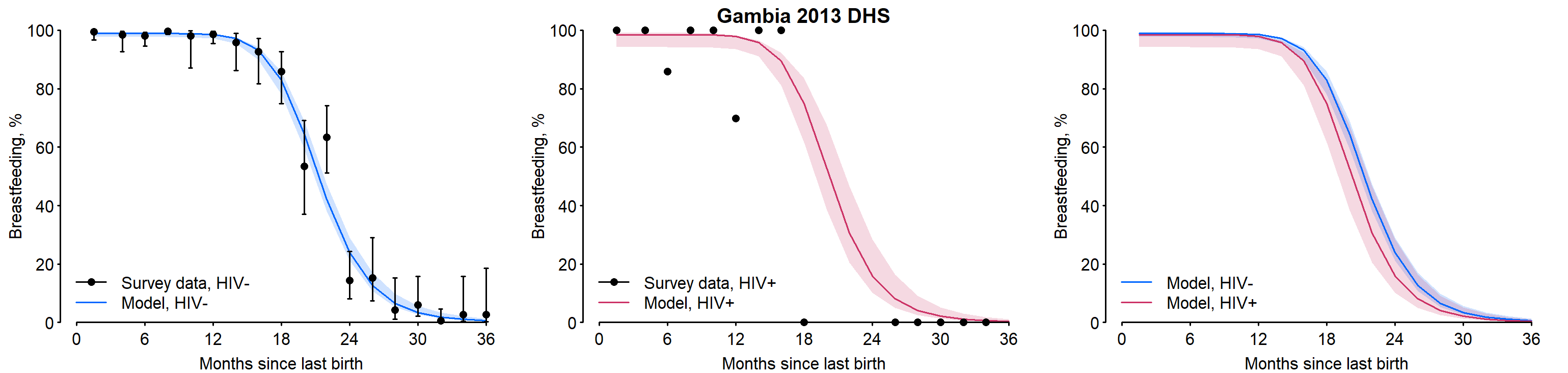

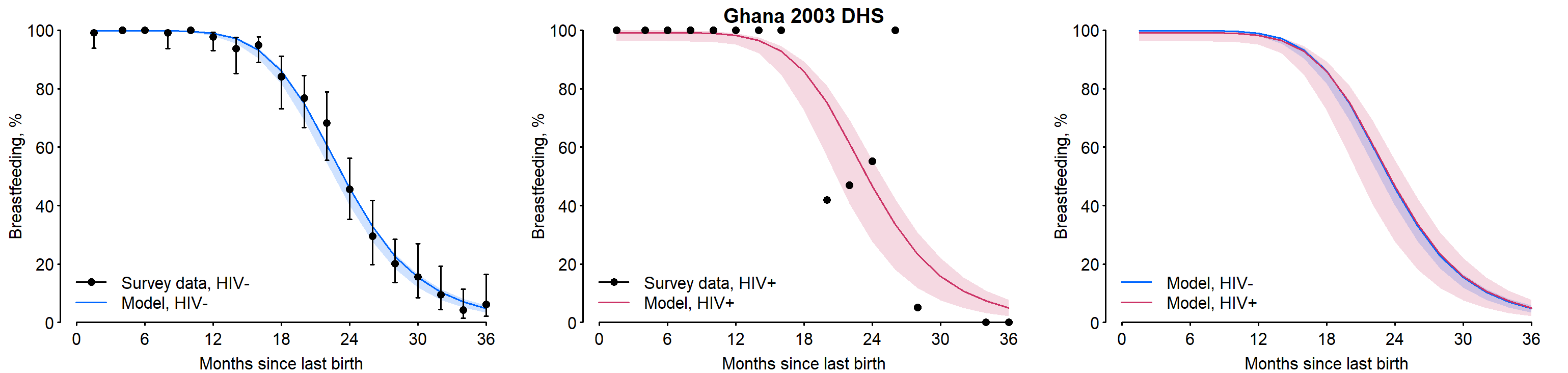

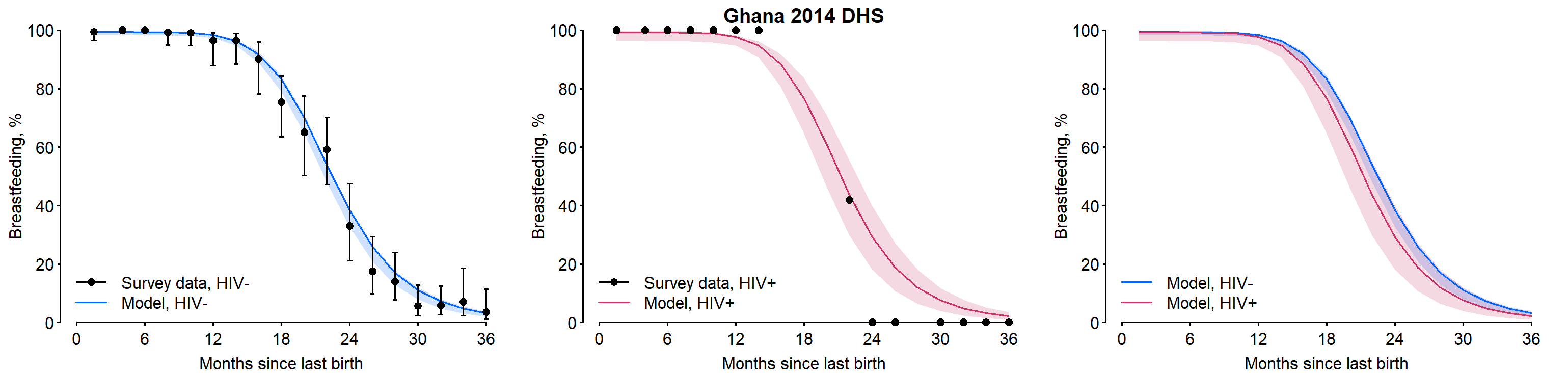

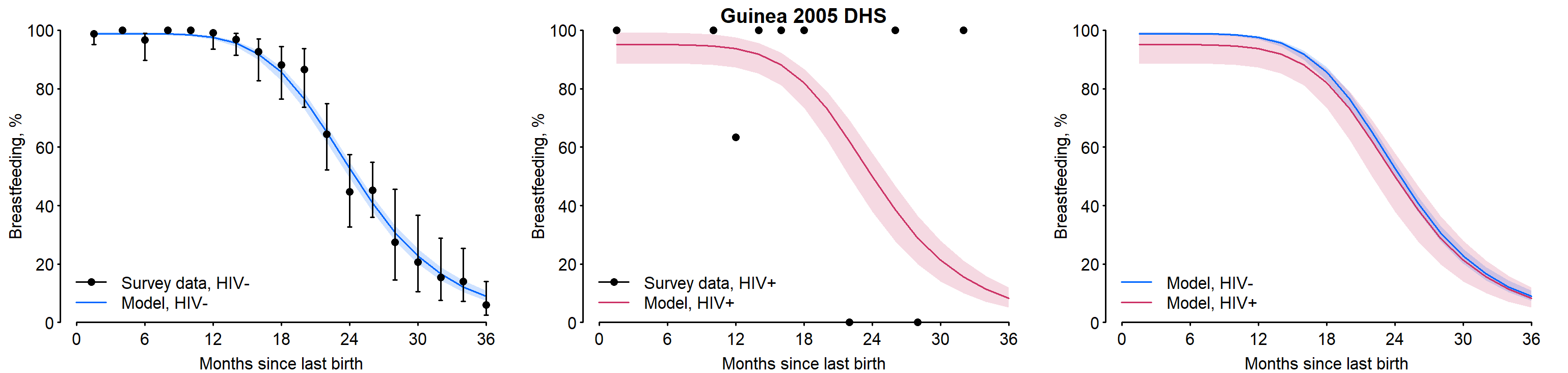

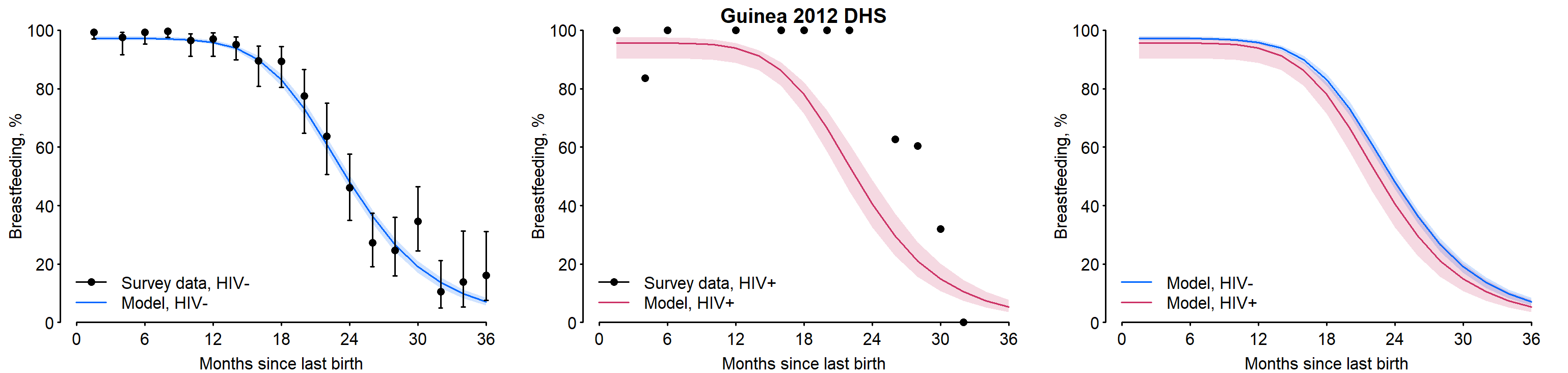

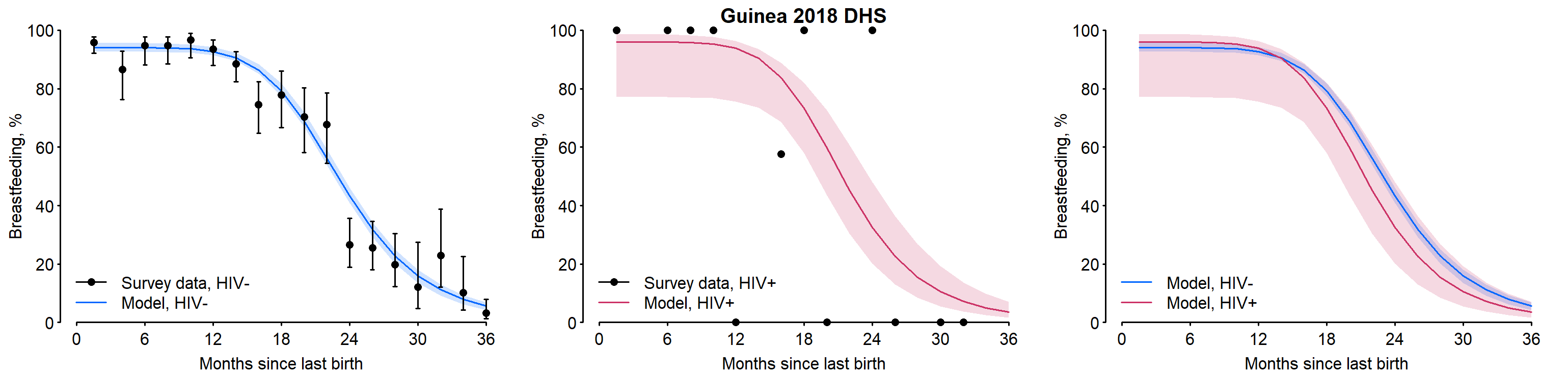

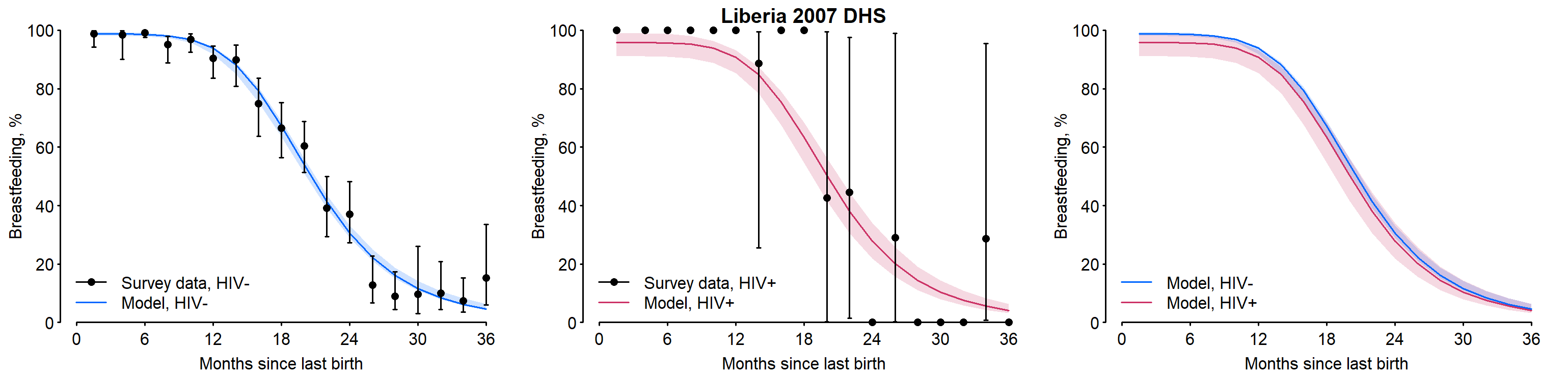

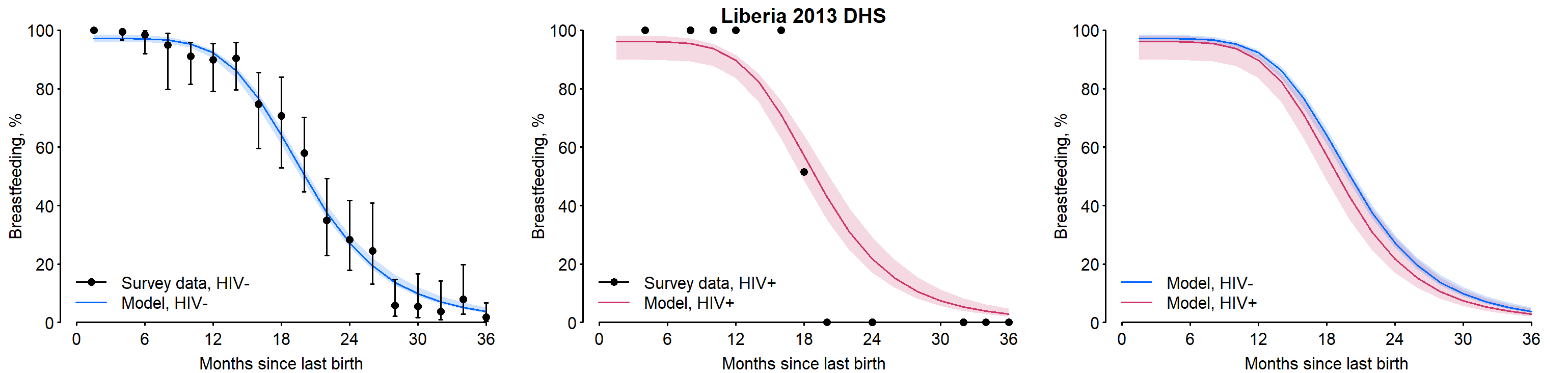

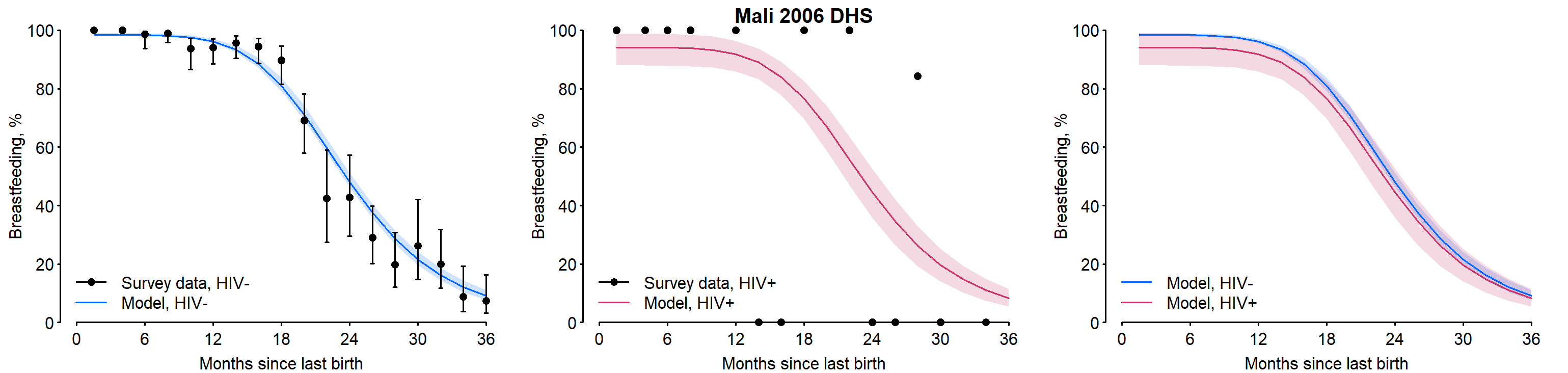

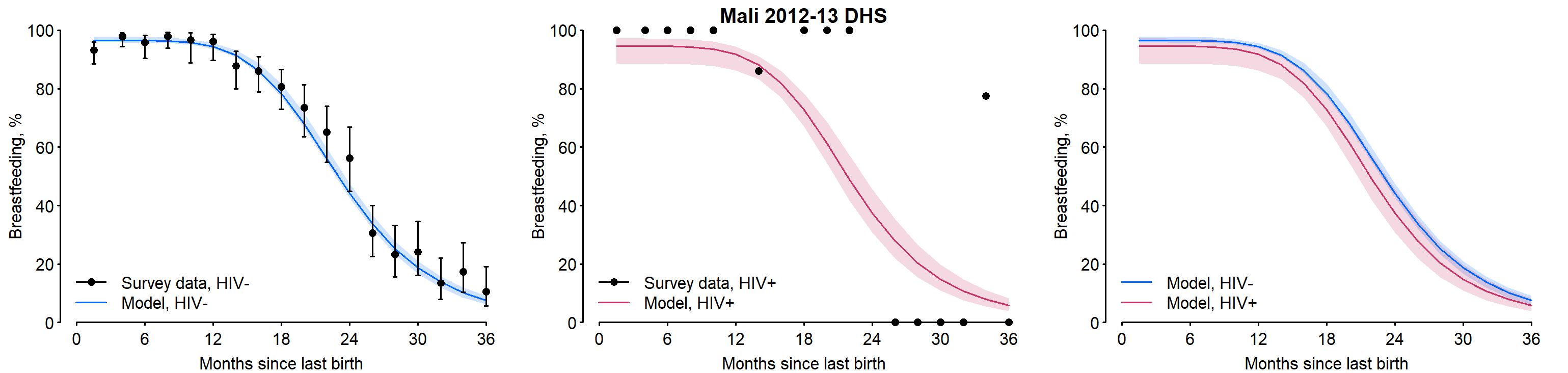

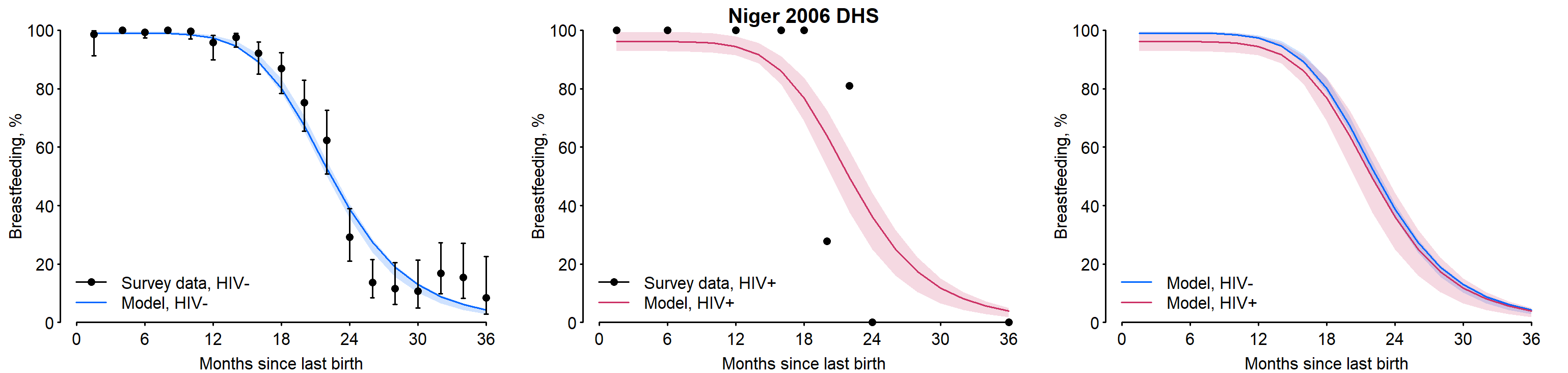

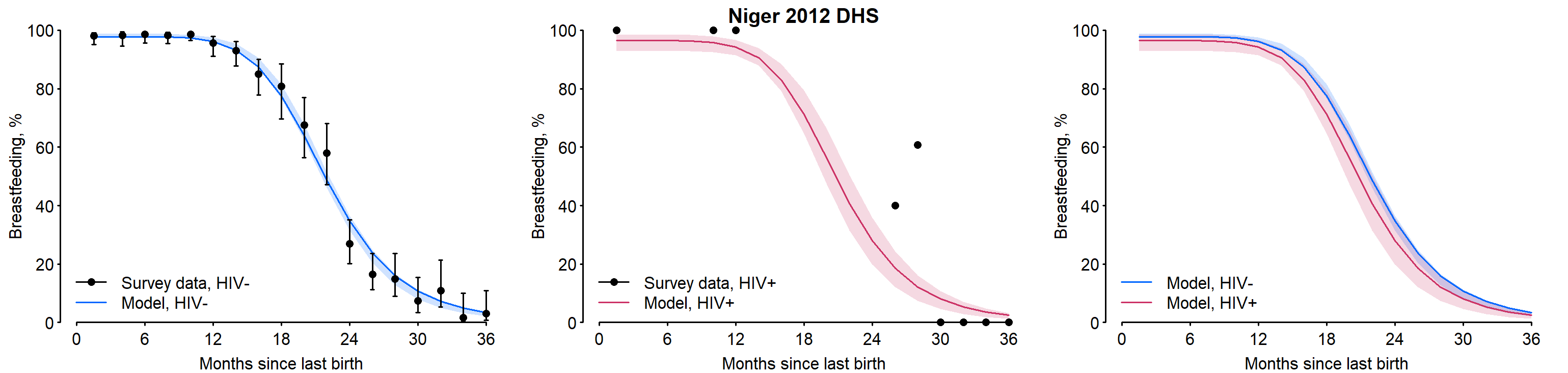

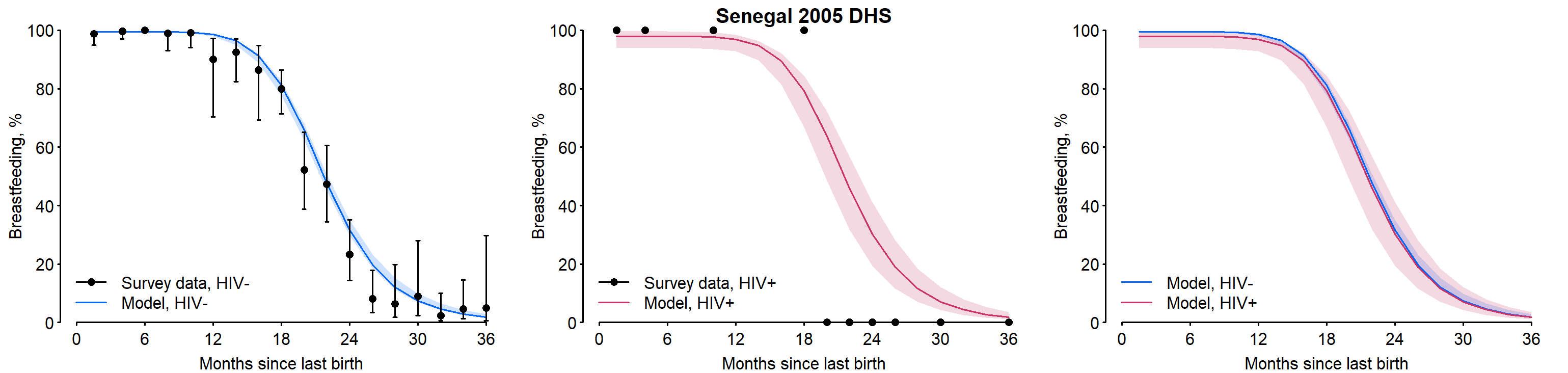

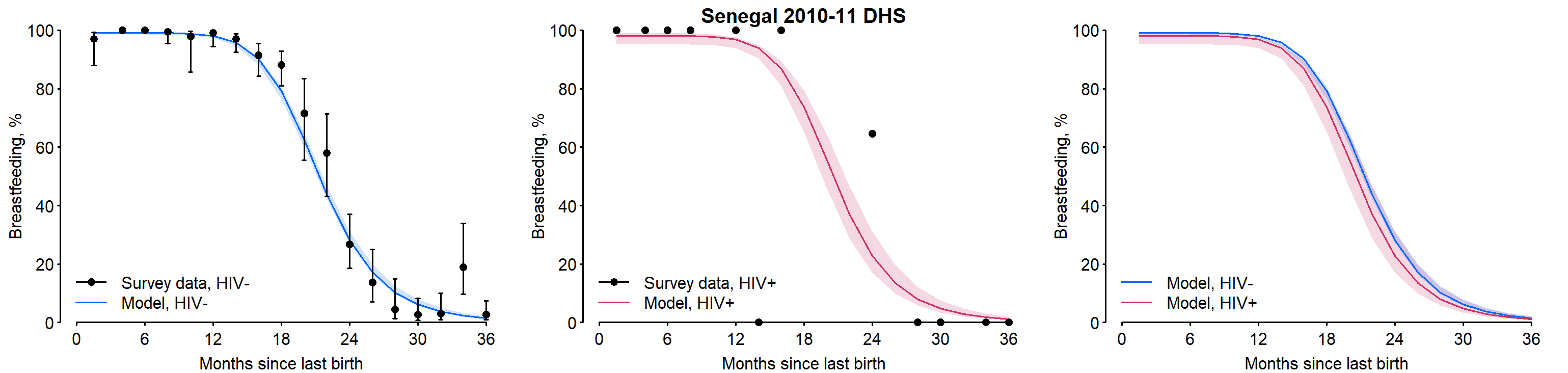

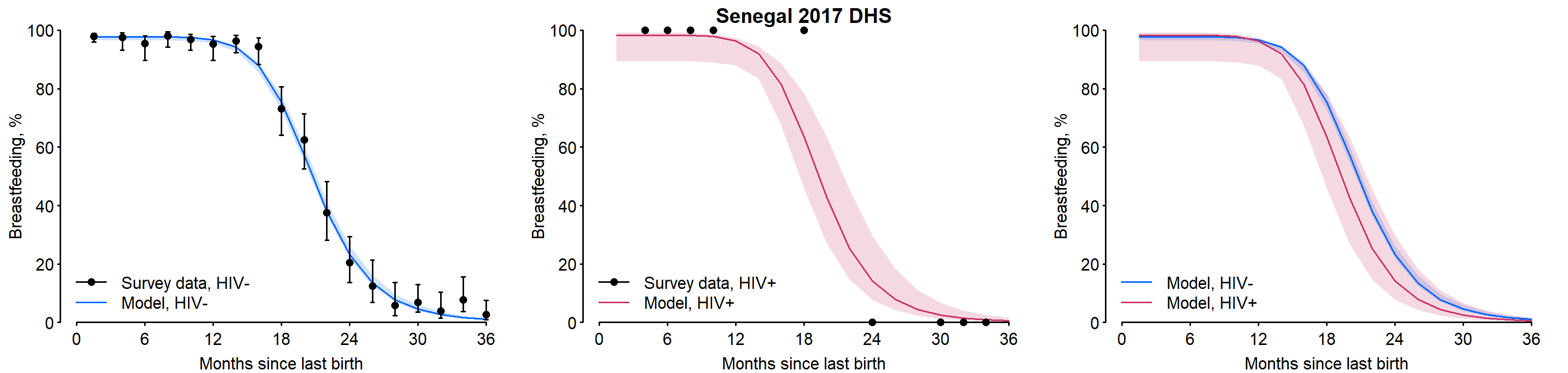

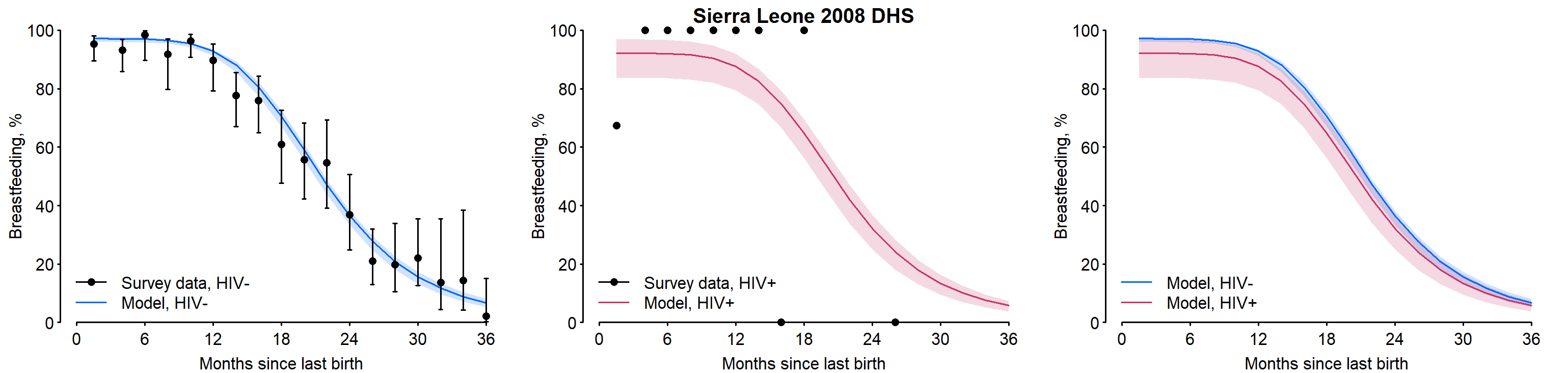

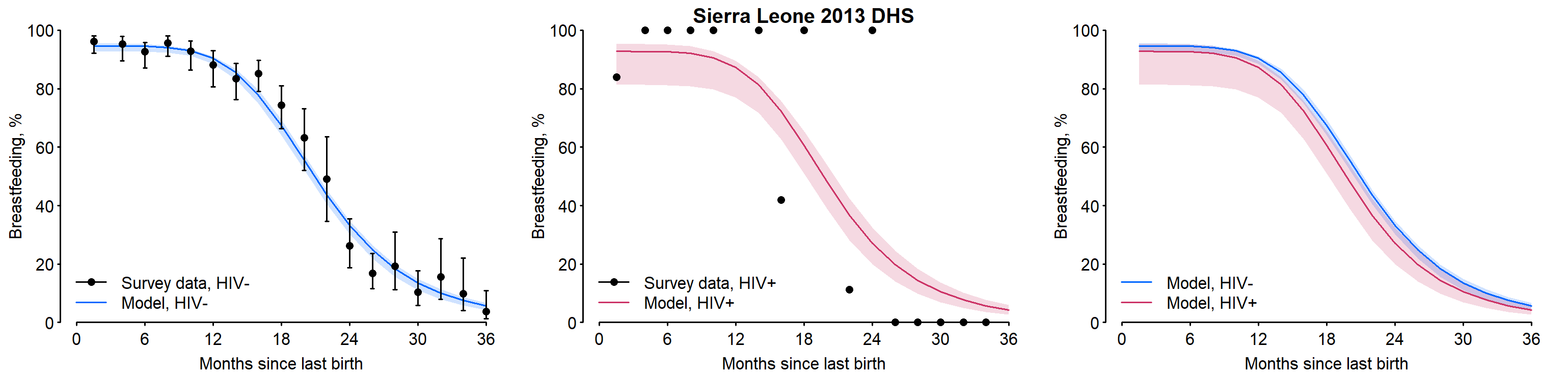

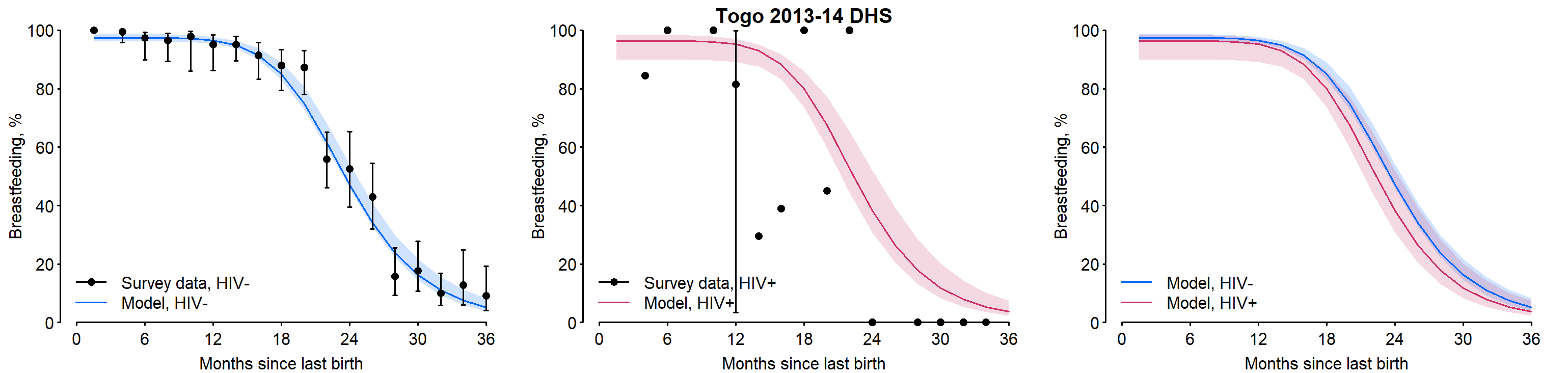


REFERENCES

1. Watson OJ, Eaton JW. rdhs: API client and dataset management for the Demographic and Health Survey (DHS) data 2019. Available from: <https://CRAN.R-project.org/package=rdhs>.

2. ICF. Demographic and Health Surveys (various) and AIDS Indicator Surveys (various). Funded by USAID. Rockville, Maryland: 2003-2018.

3. Kenya National Bureau of Statistics. Kenya AIDS Indicator Survey 2012 2018 [May 18, 2021]. Available from: <http://statistics.knbs.or.ke/nada/index.php/catalog/94>.

4. ICAP at Columbia University. PHIA Project: A Drop that Counts [cited 2021 March 3]. Available from: <https://phia.icap.columbia.edu/>.

5. Lumley T. Survey: Analysis of complex survey samples 2020 [May 17, 2021]. R package version 4.0. Available from: <https://CRAN.R-project.org/package=survey>.

We conducted a secondary analysis to assess the sensitivity of breastfeeding patterns estimated for Southern Africa to inclusion of South Africa’s 2016 DHS. Since surveys in other countries in the region are older (2006-2014), our primary analysis might conflate national and temporal differences in breastfeeding patterns. We estimated larger differences in breastfeeding practices among HIV-positive mothers when the South African survey was included in our analysis in comparison to exclusion of that survey. These differences were largest in 2005 but narrowed over time. This analysis should be updated as new survey data become available to better distinguish national and temporal effects.

We evaluated the effects of proposed model-based breastfeeding patterns on new pediatric HIV infections in Sub-Saharan Africa. We used Spectrum/AIM files from the 2018 HIV estimates round for this analysis, as some countries adopted preliminary model-based breastfeeding patterns in later rounds. We compared new pediatric infection estimates when using either original breastfeeding patterns from 2018 Spectrum/AIM files or our proposed patterns estimated among mothers living with HIV. Original inputs usually ignored maternal HIV status and assumed breastfeeding practices did not change over time.

In comparison to the original breastfeeding inputs, Spectrum/AIM estimated fewer new pediatric HIV infections throughout the sub-Saharan African epidemic when proposed breastfeeding patterns were assumed (Figure 4 in main text). At their peak in 2000, new pediatric HIV infections estimates were 2.6% lower with proposed (430,200) compared to original (441,500) inputs, while estimates in 2017 were 10.7% lower (proposed: 166,900; original: 186,900). This might overstate the difference, since countries probably would not change BF inputs in a vacuum but might adjust HIV-related fertility effects to compensate for BF changes.


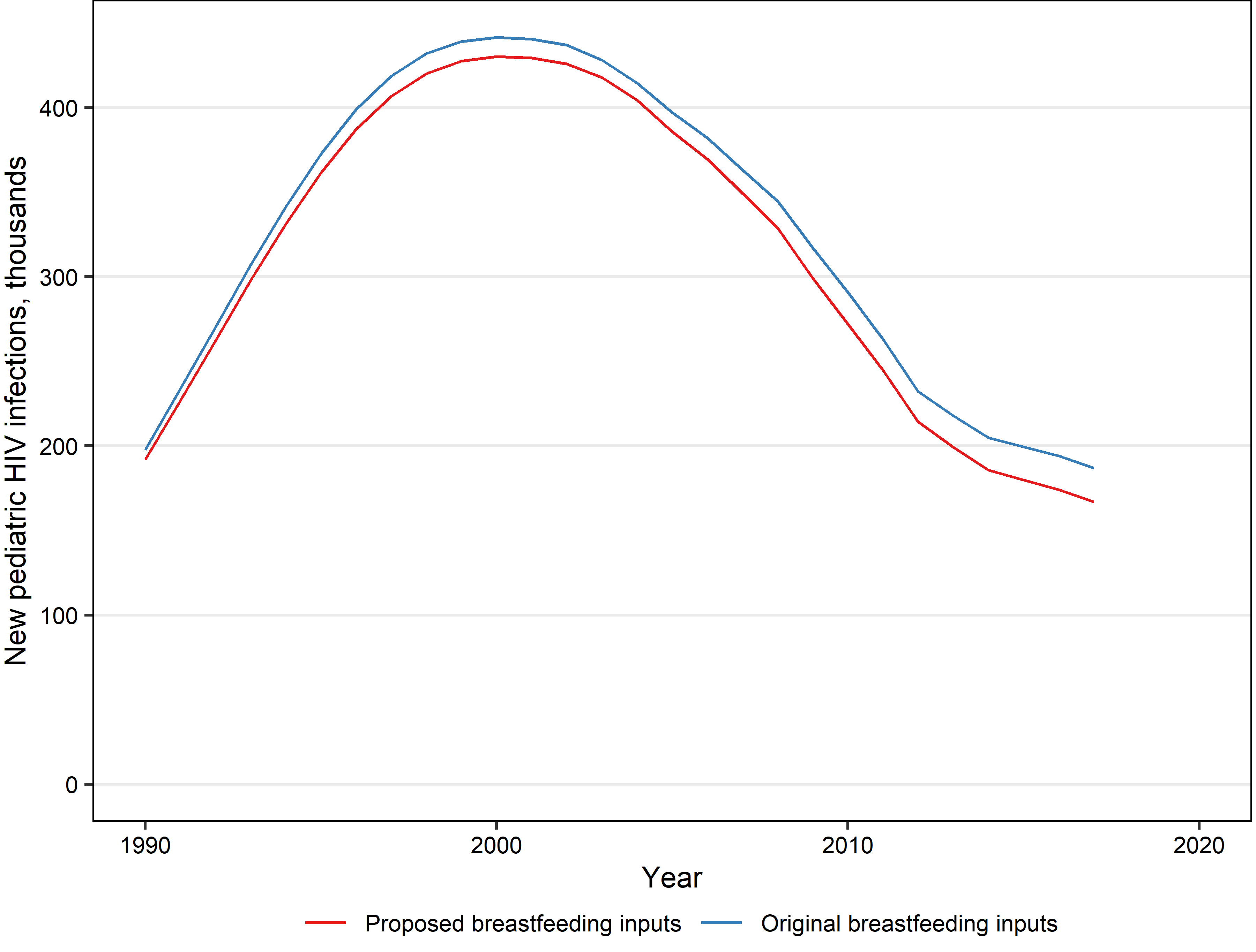


**Figure S2. New pediatric HIV infections in sub-Saharan Africa. Curves show numbers of new pediatric HIV infections estimated by Spectrum/AIM when using different breastfeeding input assumptions.**
