# Supplementary material for: Updates to the Spectrum/AIM model for the UNAIDS 2020 HIV estimates
Source: J Int AIDS Soc. 2021 Sep 21;24(Suppl 5):e25778. doi: 10.1002/jia2.25778 (PMC8454674; doi:10.1002/jia2.25778)
Supplement: Supplementary file 3 — Appendix S3. Mapping the transition from CD4 percent to CD4 count for HIV‐infected children [file JIA2-24-e25778-s003.docx]

**Supplemental Appendix 3: Mapping the transition from CD4 percent to CD4 count for HIV-infected children**

The transition probabilities shown in Table 1 of the main manuscript are plotted below, together with uncertainties and previously used transition probabilities. Confidence intervals are obtained using the method of Goodman^[[1]](#footnote-1)^.


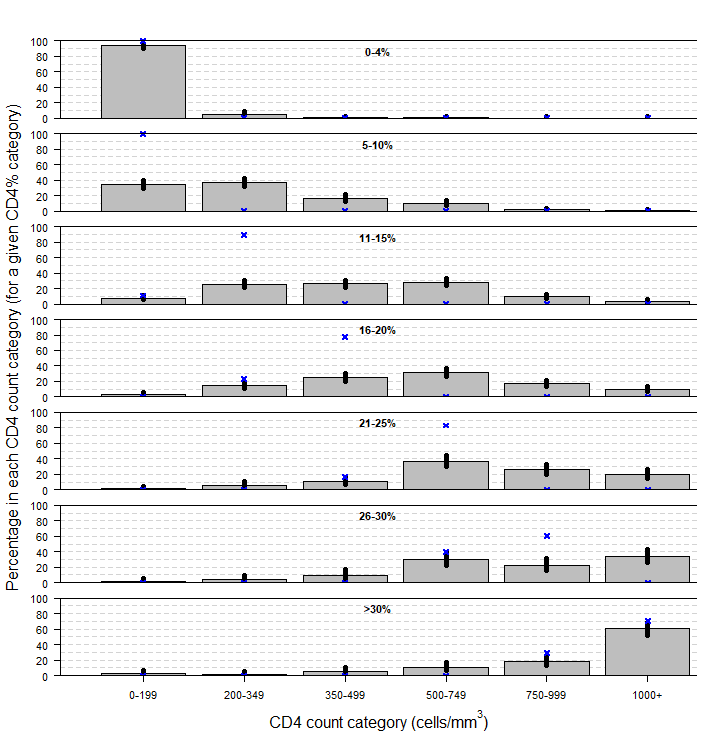


Figure 1. CD4 percentage to count transition probabilities. The percentage (y-axis) of children in each CD4 count category (x-axis) is shown, per CD4% category (the seven distinct subplots); point estimates are shown by grey bars, 95% confidence intervals by black lines; and previously used transition percentages by blue crosses.

In sensitivity analyses, we restricted children further, at the cost of a reduced sample size, to start ART (i) within 3 months of either side of their 5^th^ birthdays (n = 1208), or (ii) 6 months after their 5th birthdays (n=1309). Results remained similar – most transition probabilities varied by less than 1% (in absolute terms), with a maximum change of 9%, though with larger uncertainties.

1. Goodman LA. On simultaneous confidence intervals for multinomial proportions. Technometrics. 1965;7:247-254. [↑](#footnote-ref-1)
