# Supplementary material for: Updates to the Spectrum/AIM model for the UNAIDS 2020 HIV estimates
Source: J Int AIDS Soc. 2021 Sep 21;24(Suppl 5):e25778. doi: 10.1002/jia2.25778 (PMC8454674; doi:10.1002/jia2.25778)
Supplement: Supplementary file 4 [file JIA2-24-e25778-s004.docx]

Acknowledgements


IeDEA Asia-Pacific
The TREAT Asia Pediatric HIV Observational Database is an initiative of TREAT Asia, a program of amfAR, The Foundation for AIDS Research, with support from the US National Institutes of Health’s National Institute of Allergy and Infectious Diseases, the Eunice Kennedy Shriver National Institute of Child Health and Human Development, National Cancer Institute, National Institute of Mental Health, National Institute on Drug Abuse, the National Heart, Lung, and Blood Institute, the National Institute on Alcohol Abuse and Alcoholism, the National Institute of Diabetes and Digestive and Kidney Diseases, and the Fogarty International Center, as part of the International Epidemiology Databases to Evaluate AIDS (IeDEA; U01AI069907). The Kirby Institute is funded by the Australian Government Department of Health and Ageing, and is affiliated with the Faculty of Medicine, UNSW Australia. The content of this publication is solely the responsibility of the authors and does not necessarily represent the official views of any of the governments or institutions mentioned above.
Site investigators and cohorts
PS Ly, V Khol, National Centre for HIV/AIDS, Dermatology and STDs, Phnom Penh, Cambodia; J Tucker, New Hope for Cambodian Children, Phnom Penh, Cambodia; N Kumarasamy, E Chandrasekaran, Chennai Antiviral Research and Treatment Clinical Research Site (CART CRS), VHS-Infectious Diseases Medical Centre, VHS, Chennai, India; A Kinikar, V Mave, S Nimkar, I Marbaniang, BJ Medical College and Sassoon General Hospitals, Maharashtra, India; DK Wati, D Vedaswari, IB Ramajaya, Sanglah Hospital, Udayana University, Bali, Indonesia; N Kurniati, D Muktiarti, Cipto Mangunkusumo – Faculty of Medicine Universitas Indonesia, Jakarta, Indonesia; SM Fong, M Lim, F Daut, Hospital Likas, Kota Kinabalu, Malaysia; NK Nik Yusoff, P Mohamad, Hospital Raja Perempuan Zainab II, Kelantan, Malaysia; TJ Mohamed, MR Drawis, Department of Pediatrics, Women IeDEA global funding acknowledgements Version 16 December 2020 4 and Children Hospital Kuala Lumpur, Kuala Lumpur, Malaysia; R Nallusamy, KC Chan, Penang Hospital, Penang, Malaysia; T Sudjaritruk, V Sirisanthana, L Aurpibul, Department of Pediatrics, Faculty of Medicine, and Research Institute for Health Sciences, Chiang Mai University, Chiang Mai, Thailand; P Ounchanum, R Hansudewechakul, S Denjanta, A Kongphonoi, Chiangrai Prachanukroh Hospital, Chiang Rai, Thailand; P Lumbiganon, P Kosalaraksa, P Tharnprisan, T Udomphanit, Division of Infectious Diseases, Department of Pediatrics, Faculty of Medicine, Khon Kaen University, Khon Kaen, Thailand; G Jourdain, PHPT-IRD UMI 174 (Institut de recherche pour le développement and Chiang Mai University), Chiang Mai, Thailand; T Puthanakit, S Anugulruengkit, W Jantarabenjakul, R Nadsasarn, Department of Pediatrics and Center of Excellence for Pediatric Infectious Diseases and Vaccines, Faculty of Medicine, Chulalongkorn University, Bangkok, Thailand; K Chokephaibulkit, K Lapphra, W Phongsamart, S Sricharoenchai, Department of Pediatrics, Faculty of Medicine Siriraj Hospital, Mahidol University, Bangkok, Thailand; KH Truong, QT Du, CH Nguyen, Children’s Hospital 1, Ho Chi Minh City, Vietnam; VC Do, TM Ha, VT An Children’s Hospital 2, Ho Chi Minh City, Vietnam; LV Nguyen, DTK Khu, AN Pham, LT Nguyen, National Hospital of Pediatrics, Hanoi, Vietnam; ON Le, Worldwide Orphans Foundation, Ho Chi Minh City, Vietnam; AH Sohn, JL Ross, T Suwanlerk, TREAT Asia/amfAR - The Foundation for AIDS Research, Bangkok, Thailand; MG Law, A Kariminia, The Kirby Institute, UNSW Sydney, NSW, Australia.


Caribbean, Central and South America network for HIV epidemiology (CCASAnet)
This work was supported by the NIH-funded Caribbean, Central and South America network for HIV epidemiology (CCASAnet), a member cohort of the International Epidemiologic Databases to Evaluate AIDS (leDEA) (U01AI069923). The content is solely the responsibility of the authors and does not necessarily represent the official views of the National Institutes of Health. This award is funded by the following institutes: the National Institute of Allergy and Infectious Diseases (NIAID), the Eunice Kennedy Shriver National Institute of Child Health and Human Development (NICHD), the National Cancer Institute (NCI), the National Institute of Mental Health (NIMH), the National Institute on Drug Abuse (NIDA), the National Heart, Lung, and Blood Institute (NHLBI), the National Institute on Alcohol Abuse and Alcoholism (NIAAA), the National Institute of Diabetes and Digestive and Kidney Diseases (NIDDK), the Fogarty International Center (FIC), and the National Library of Medicine (NLM).
Site investigators and cohorts
Fundación Huésped, Argentina: Pedro Cahn, Carina Cesar, Valeria Fink, Omar Sued, Emanuel Dell’Isola, Hector Perez, Jose Valiente, Cleyton Yamamoto.
Instituto Nacional de Infectologia-Fiocruz, Brazil: Beatriz Grinsztejn, Valdilea Veloso, Paula Luz, Raquel de Boni, Sandra Cardoso Wagner, Ruth Friedman, Ronaldo Moreira.
Universidade Federal de Minas Gerais, Brazil: Jorge Pinto, Flavia Ferreira, Marcelle Maia.
Universidade Federal de São Paulo, Brazil: Regina Célia de Menezes Succi, Daisy Maria Machado, Aida de Fátima Barbosa Gouvêa.
Fundación Arriarán, Chile: Marcelo Wolff, Claudia Cortes, Maria Fernanda Rodriguez, Gladys Allendes.
Les Centres GHESKIO, Haiti: Jean William Pape, Vanessa Rouzier, Adias Marcelin, Christian Perodin. Hospital Escuela Universitario, Honduras: Marco Tulio Luque.
Instituto Hondureño de Seguridad Social, Honduras: Denis Padgett.
Instituto Nacional de Ciencias Médicas y Nutrición Salvador Zubirán, Mexico: Juan Sierra Madero, Brenda Crabtree Ramirez, Paco Belaunzaran, Yanink Caro Vega.
Instituto de Medicina Tropical Alexander von Humboldt, Peru: Eduardo Gotuzzo, Fernando Mejia, Gabriela Carriquiry.
Vanderbilt University Medical Center, USA: Catherine C McGowan, Bryan E Shepherd, Timothy Sterling, Karu Jayathilake, Anna K Person, Peter F Rebeiro, Jessica Castilho, Stephany N Duda, Fernanda Maruri, Hilary Vansell, Cathy Jenkins, Ahra Kim, Sarah Lotspeich.


East Africa IeDEA
Research reported in this publication was supported by the National Institute Of Allergy And Infectious Diseases (NIAID), Eunice Kennedy Shriver National Institute Of Child Health & Human Development (NICHD), National Institute On Drug Abuse (NIDA), National Cancer Institute (NCI), and the National Institute of Mental Health (NIMH), National Institute of Diabetes and Digestive and Kidney Diseases (NIDDK) , Fogarty International Center (FIC), National Heart, Lung, and Blood Institute (NHLBI) , in accordance with the regulatory requirements of the National Institutes of Health under Award Number U01AI069911East Africa IeDEA Consortium. The content is solely the responsibility of the authors and does not necessarily represent the official views of the National Institutes of Health.
Site investigators, cohorts, data managers
Diero L, Ayaya S, Sang E, MOI University, AMPATH Plus, Eldoret, Kenya; Bukusi E, Edwin Mulwa, George Nyanaro, KEMRI (Kenya Medical Research Institute), Kisumu, Kenya; Charles Kasozi , Mathew Ssemakadde, Masaka Regional Referral Hospital, Masaka, Uganda; Mwebesa Bosco Bwana, Winnie Muyindike, Helen Byakwaga Michael Kanyesigye, Mbarara University of Science and Technology (MUST), Mbarara, Uganda; Barbara Castelnuovo, Aggrey Semeere,; John Michael Matovu, Infectious Diseases Institute (IDI), Mulago, Uganda; Fred Nalugoda, Francis X. Wasswa, Rakai Health Sciences Program, Kalisizo, Uganda; Paul Kazyoba, Mary Mayige, (NIMR), Dar es Salaam, Tanzania; Rita Elias Lyamuya, Francis Mayanga, Morogoro Regional Hospital, Morogoro, Tanzania; Kapella Ngonyani, Jerome Lwali, Tumbi Regional Hospital, Pwani, Tanzania; Mark Urassa, Charles Nyaga, Richard Machemba, National Institute for Medical Research (NIMR), Kisesa HDSS, Mwanza, Tanzania; Kara Wools-Kaloustian, Constantin Yiannoutsos, Beverly Musick, Indiana University School of Medicine, Indiana University, Indianapolis, IN, USA; Batya Elul, Columbia University, New York City, NY, USA; Rachel Vreeman (Mt. Sinai, New York, USA) Jennifer Syvertsen, (University of California Riverside, California, USA; Rami Kantor, Brown University/Miriam Hospital, Providence, RI, USA; Jeffrey Martin, Megan Wenger, Craig Cohen, Jayne Kulzer, University of California, San Francisco, CA, USA;


IeDEA Southern Africa
Research reported in this publication was supported by the U.S. National Institutes of Health’s National Institute of Allergy and Infectious Diseases, the Eunice Kennedy Shriver National Institute of Child Health and Human Development, the National Cancer Institute, the National Institute of Mental Health, the National Institute on Drug Abuse, the National Heart, Lung, and Blood Institute, the National Institute on Alcohol Abuse and Alcoholism, the National Institute of Diabetes and Digestive and Kidney Diseases and the Fogarty International Center under Award Number U01AI069924. The content is solely the responsibility of the authors and does not necessarily represent the official views of the National Institutes of Health.
Site investigators and cohorts
Gary Maartens, Aid for AIDS, South Africa; Carolyn Bolton, Centre for Infectious Disease Research in Zambia (CIDRZ), Zambia; Robin Wood, Gugulethu (Desmond Tutu HIV Centre), South Africa; Nosisa Sipambo, Harriet Shezi Children’s Clinic, South Africa; Frank Tanser, Hlabisa (Africa Health Research Institute), South Africa; Andrew Boulle, Khayelitsha ART Programme, South Africa; Geoffrey Fatti, Kheth’Impilo AIDS Free Living, South Africa; Sam Phiri, Lighthouse Trust, Malawi; Elvira Singh, National Cancer Registry (National Health Laboratory Service), South Africa; Cleophas Chimbetete, Newlands Clinic (Ruedi Luethy Foundation Zimbabwe), Zimbabwe; Karl Technau, Rahima Moosa Mother and Child Hospital, South Africa; Brian Eley, Red Cross War Memorial Children’s Hospital, South Africa; Josephine Muhairwe, SolidarMed Lesotho; Idivino Rafael, SolidarMed Mozambique; Cordelia Kunzekwenyika, SolidarMed Zimbabwe, Matthew P Fox, Themba Lethu Clinic, South Africa; Hans Prozesky, Tygerberg Hospital, South Africa.
Data centers
Nina Anderegg, Marie Ballif, Benedikt Christ, Cam Ha Dao Ostinelli, Matthias Egger, Lukas Fenner, Andreas Haas, Anthony Hauser, Stefanie Hossmann, Serra Lem, Catrina Mugglin, Radoslaw Panczak, Eliane Rohner, Julien Riou, Veronika Skrivankova, Lilian Smith, Katayoun Taghavi, Per von Groote, Gilles Wandeler, Elizabeth Zaniewski, Kathrin Zürcher, Institute of Social and Preventive Medicine, University of Bern, Switzerland; Kim Anderson, Andrew Boulle, Morna Cornell, Mary-Ann Davies, Victoria Iyun, Leigh Johnson, Reshma Kassanjee, Kathleen Kehoe, Mmamapudi Kubjane, Nicola Maxwell, Patience Nyakato, Gem Patten, Mpho Tlali, Priscilla Tsondai, Renee de Waal, School of Public Health and Family Medicine, University of Cape Town, South Africa.


IeDEA West Africa
Research reported in this publication was supported by the US National Institutes of Health (NIAID, NICHD, NCI and NIMH) under Award Number U01AI069919 (PI: Dabis). The content is solely the responsibility of the authors and does not necessarily represent the official views of the National Institutes of Health.
Site investigators and cohorts
Adult cohorts: Marcel Djimon Zannou, CNHU, Cotonou, Benin; Armel Poda, CHU Souro Sanou, Bobo Dioulasso, Burkina Faso; Fred Stephen Sarfo & Komfo Anokeye Teaching Hospital, Kumasi, Ghana; Eugene Messou, ACONDA CePReF, Abidjan, Cote d’Ivoire; Henri Chenal, CIRBA, Abidjan, Cote d’Ivoire; Kla Albert Minga, CNTS, Abidjan, Cote d’Ivoire; Emmanuel Bissagnene, & Aristophane Tanon, CHU Treichville, Cote d’Ivoire; Moussa Seydi, CHU de Fann, Dakar, Senegal; Akessiwe Akouda Patassi, CHU Sylvanus Olympio, Lomé, Togo. Pediatric cohorts: Sikiratou Adouni Koumakpai-Adeothy,_CNHU, Cotonou, Benin; Lorna Awo Renner, Korle Bu Hospital, Accra, Ghana; Sylvie Marie N’Gbeche, ACONDA CePReF, Abidjan, Ivory Coast; Clarisse Amani Bosse, ACONDA_MTCT+, Abidjan, Ivory Coast; Kouadio Kouakou, CIRBA, Abidjan, Cote d’Ivoire; Madeleine Amorissani Folquet, CHU de Cocody, Abidjan, Cote d’Ivoire; François Tanoh Eboua, CHU de Yopougon, Abidjan, Cote d’Ivoire; Fatoumata Dicko Traore, Hopital Gabriel Toure, Bamako, Mali; Elom Takassi, CHU Sylvanus Olympio, Lomé,Togo
Coordinating and data centers
François Dabis, Elise Arrive, Eric Balestre, Renaud Becquet, Charlotte Bernard, Shino Chassagne Arikawa, Alexandra Doring, Antoine Jaquet, Karen Malateste, Elodie Rabourdin, Thierry Tiendrebeogo, ADERA, Isped & INSERM U1219, Bordeaux, France. Sophie Desmonde, Julie Jesson, Valeriane Leroy, Inserm 1027, Toulouse, France Didier Koumavi Ekouevi, Jean-Claude Azani, Patrick Coffie, Abdoulaye Cissé, Guy Gnepa, Apollinaire Horo, Christian Kouadio, Boris Tchounga, PACCI, CHU Treichville, Abidjan, Côte d’Ivoire
